# Antidepressant-like activity and safety profile evaluation of 1*H*-imidazo[2,1-*f*]purine-2,4(3H,8H)-dione derivatives as 5-HT_1A_ receptor partial agonists

**DOI:** 10.1371/journal.pone.0237196

**Published:** 2020-08-07

**Authors:** Anna Partyka, Agnieszka Zagórska, Magdalena Kotańska, Maria Walczak, Magdalena Jastrzębska-Więsek, Joanna Knutelska, Marek Bednarski, Monika Głuch-Lutwin, Barbara Mordyl, Paulina Janiszewska, Anna Wesołowska

**Affiliations:** 1 Department of Clinical Pharmacy, Faculty of Pharmacy, Jagiellonian University Medical College, Krakow, Poland; 2 Department of Medicinal Chemistry, Faculty of Pharmacy, Jagiellonian University Medical College, Krakow, Poland; 3 Department of Pharmacological Screening, Faculty of Pharmacy, Jagiellonian University Medical College, Krakow, Poland; 4 Chair and Department of Toxicology, Faculty of Pharmacy, Jagiellonian University Medical College, Krakow, Poland; 5 Department of Pharmacobiology, Faculty of Pharmacy, Jagiellonian University Medical College, Krakow, Poland; Western University of Health Sciences, UNITED STATES

## Abstract

Current antidepressant therapy has several disadvantages related to the properties of antidepressants. Considering their unfavourable features, the process of searching for new antidepressant drugs with better safety and tolerability requires consistent efforts and many complementary studies. Serotonin 5-HT_1A_ receptor is considered as an interesting target of antidepressant therapy. In the present study, the intrinsic activity at different signaling pathways coupled to serotonin 5-HT_1A_ receptor, antidepressant-like and pharmacokinetic properties, and the safety profile of two novel imidazopurine-2,4-dione derivatives, namely compounds AZ-853 (8-(4-(4-(2-fluorophenyl)piperazin-1-yl)butyl)-1,3-dimethyl-1H- imidazo[2,1-f]purine-2,4(3H,8H)-dione) and AZ-861 (1,3-dimethyl-8-(4-(4-(3-(trifluoromethyl)phenyl)piperazin-1-yl)butyl)-1H-imidazo[2,1-f]purine-2,4(3H,8H)-dione), were studied in animal models through *in vitro* and *in vivo* experiments. We demonstrated that AZ-853 and AZ-861, which structurally differ by one substituent and its placement in the phenyl ring, showed varied functional, pharmacological, and pharmacokinetic properties as well as side effect profiles. AZ-861 exhibited stronger agonistic action in all functional assays. After acute and repeated administration in mice, both compounds showed antidepressant-like activity in the forced swim test, which was partially mediated by 5-HT_1A_ receptor activation. AZ-853 showed a more potent antidepressant-like effect, presumably due to its better penetration into brain structures. Both compounds did not show anticholinergic properties, but after repeated administration, they induced weak sedation and lipid metabolism disturbances without affecting serum glucose level. The stronger α_1_-adrenolytic effect of AZ-853 is responsible for decreased systolic blood pressure, and in contrast to AZ-861, AZ-853 induced weight gain in mice. The interesting comparative pharmacological profiles of AZ-853 and AZ-861 encourage to conduct further experiments to fully understand their mechanisms and differences in action.

## Introduction

World Health Organization (WHO) has predicted that major depressive disorder (MDD) will become the second cause of illness worldwide by 2030 [1]. This implies an inevitable increase in the consumption of antidepressant drugs (ADDs). In Europe, the use of ADDs has increased substantially during the last few decades, and between 2001 and 2010, the use of ADDs increased by an average of 20% per year [2]. The main disadvantage of antidepressant therapy is side effects (SEs) of ADDs. SEs not only generate health, social, and economic costs, but they are also some of the most common factors responsible for nonadherence and premature discontinuation of treatment [3].

Most ADDs may increase weight in a significant percentage of patients receiving acute and/or maintenance therapy. Tricyclic antidepressants (TCAs), starting with amitriptyline, are the most weight gain-inducing compounds among the ADDs during both short- and long-term treatments. Mirtazapine is also associated with significant weight gain during acute and maintenance therapy. The short-term usage of selective serotonin reuptake inhibitors (SSRIs), serotonin and noradrenaline reuptake inhibitors (SNRIs), moclobemide (a reversible inhibitor of monoamine oxidase A), and bupropion (a dopamine and noradrenaline reuptake inhibitor) induces weight loss. However, over the long-term period, bupropion alone maintains its influence on weight. Among SSRIs, paroxetine is considered to cause the greatest weight increase during maintenance therapy [4]. TCAs and mirtazapine may also affect metabolic parameters such as plasma lipid profile. Moreover, ADDs, mainly SSRIs and TCAs, increase the risk of diabetes mellitus [3].

Cardiovascular SEs are another group of adverse effects caused by antidepressant therapy. TCAs that cause life-threatening arrhythmias and orthostatic hypotension have the highest potential of inducing such SEs [5,6]. The new generation of ADDs also has clinically important cardiovascular effects. Among SSRIs, citalopram appears to most significantly prolong QT interval, while paroxetine seems to be most frequently associated with orthostatic hypotension. SNRIs may cause clinically significant increase in diastolic blood pressure [3,5,6]. The majority of adverse effects of TCAs are due to blockade of histamine H_1_ (sedation, weight gain, hypotension), muscarinic (dry mouth, urinary retention, memory dysfunction), and adrenergic α_1_ (orthostatic hypotension, dizziness) receptors. Most of the new generation ADDs are practically devoid of blocking effects for neurotransmitter receptors [7].

Since 1950s, when the introduction of TCAs and monoamine oxidase inhibitors revolutionized the treatment of depression, tremendous progress has been made in the development of better tolerated ADDs. Nevertheless, the existing disadvantages of antidepressant therapy necessitate consistent efforts to develop ADDs with better safety and tolerability profile.

**Fig 1:**
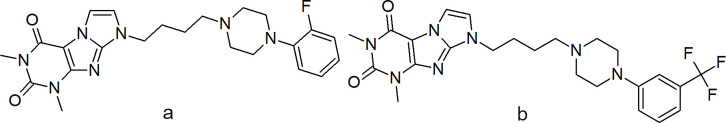
Chemical structures of the studied compounds AZ-853 (a) and AZ-861 (b).

The present study aimed to investigated the antidepressant-like properties of two novel derivatives of imidazopurine-2,4-dione, namely compounds AZ-853 (8-(4-(4-(2-fluorophenyl)piperazin-1-yl)butyl)-1,3-dimethyl-1H- imidazo[2,1-f]purine-2,4(3H,8H)-dione) and AZ-861 (1,3-dimethyl-8-(4-(4-(3-(trifluoromethyl)phenyl)piperazin-1-yl)butyl)-1H-imidazo[2,1-f]purine-2,4(3H,8H)-dione) (Fig 1), in the forced swim test (FST) in mice after single and repeated administration. In our earlier paper, we demonstrated that AZ-853 and AZ-861 possessed high affinity for serotonergic 5-HT_1A_ receptor (5-HT_1A_R) (K_i_ = 0.6 nM and K_i_ = 0.2 nM, respectively) and moderate/low affinity for serotonergic 5-HT_7_ receptor (5-HT_7_R) (K_i_ = 508 nM and K_i_ = 136 nM, respectively). The compounds act as potent 5-HT_1A_R antagonists in CHO-K1 cells in the Ca^2+^ mobilisation assay (K_b_ = 2.7 nM and K_b_ = 1.4 nM, respectively) [8]. Given that 5-HT_1A_R is associated with several different transduction pathways and that 5-HT_1A_R ligands may activate them to varying degrees or even act as an agonist in one of them and as an antagonist in other pathways, in the present study, we investigated the ability of both the selected compounds to regulate other signaling pathways coupled to 5-HT_1A_R (i.e., extracellular-regulated kinase 1/2 phosphorylation (pERK1/2), adenylyl cyclase inhibition (cAMP), β-arrestin recruitment) and subsequently determined whether their 5-HT_1A_R intrinsic activity is responsible for the effects observed in FST. As pharmacokinetic studies are quite important for developing new bioactive compounds, we examined the pharmacokinetic properties of AZ-853 and AZ-861 through a single intraperitoneal (*i*.*p*.) administration. We also performed a battery of tests to investigate the safety profile of the tested compounds, particularly their potency to induce cardiologic and metabolic SEs.

## Materials and methods

### Functional assays for the 5-HT1AR *in vitro*

Test and reference compounds were dissolved in DMSO at a concentration of 10 mM. E_max_ values were defined as the response of the ligand expressed as a percentage of the maximal response elicited by serotonin, determined by nonlinear regression using GraphPad Prism 6.0 software.

#### Cells

CHO-K1 cell line transfected with 5-HT1A receptor, were commercially obtained from Perkin Elmer (Cat. No. ES-310-C, Lot Number M2W-C1). Cells were cultured according to manufacturer’s instruction. Cell Line Development: Proprietary bicistronic expression plasmid containing the sequence coding for the human serotonin 5-HT1A receptor was transfected in CHO-K1 cells. Geneticin-resistant clones were obtained by limit dilution and compared for receptor expression levels using a radioligand binding assay. The clone with the highest receptor expression level was selected for characterization in binding and functional assays. DNA Sequence: Identical to coding sequence of GenBank M83181.1. with the exception of a silent mutation in codon # 117 (GTG becomes GTC, both coding for a Val). Corresponding Protein Sequence: Identical to GenBank NP_000515.2. Receptor expression level (BMAX): Estimated to be 3.7 ± 2.3 pmol/mg protein, using [3H] 8-OHDPAT. This cell line tested negative for mycoplasma.

U2OS HTR1A-bla cell line was commercially obtained from Life Technologies (Cat. No. K1857PPS, Lot Number 1835727). Cells were cultured according to manufacturer’s instruction. Tango™ HTR1A-bla U2OS cells contain the human Serotonin Type 1A receptor linked to a TEV protease site and a Gal4-VP16 transcription factor stably integrated into the Tango™ GPCR-bla U2OS parental cell line. This parental cell line stably expresses a beta-arrestin/TEV protease fusion protein and the beta-lactamase reporter gene under the control of a UAS response element. The Tango™ HTR1A-bla U2OS cells have been functionally validated for a response to 5-CT. This cell line tested negative for mycoplasma.

#### Extracellular signal-Regulated Kinase 1/2 (ERK1/2) phosphorylation

The CHO-K1 5HT_1A_R cells were cultured in the medium Advanced DMEM/F12 with 1% fetal bovine serum (FBS) dialyzed, 400 μg/mL G-418, 4 mM l-glutamine. In the experiment, the cells were plated at 50 000 cells/well of a 96-well tissue culture plate and grown for 7 hours, then were starved (DMEM/F12 with 0.1% BSA (immunoglobulin- and protease-free) for 12 h. The serial dilutions of compounds were added and incubated for 15 min at 37°C. The CHO-K1 5HT_1A_R cells were tested for agonist-induced ERK1/2-phosphorylation using the SureFire ERK1/2-Phosphorylation Alpha LISA assay kit according to the manufacturer’s instruction (PerkinElmer). After incubation, the assay plate was measured in an EnVision multifunction plate reader (PerkinElmer, USA).

#### cAMP inhibition

For 5-HT_1A_R, adenylyl cyclase activity was determined using cryopreserved CHO-K1 cells with expression of the human serotonin 5-HT_1A_R, where the plasmid containing the coding sequence was transfected in. The cells were cultured under selective conditions (400 μg/mL Geneticin G418). Thawed cells were resuspended in stimulation buffer (HBSS, 5 mM HEPES, 0.5 IBMX, and 0.1% BSA at pH 7.4) at 2 × 10^5^ cells/mL. The same volume (10 μL) of cell suspension was added to tested compounds with 10 μM forskolin. Cell stimulation was performed for 40 min at room temperature. After incubation, cAMP measurements were performed with homogeneous TR-FRET immunoassay using the LANCE Ultra cAMP kit (PerkinElmer, USA) according to the manufacturer’s instruction (PerkinElmer). The TR-FRET signal was read on an EnVision microplate reader (PerkinElmer, USA).

#### β-Arrestin recruitment

The HTR1A-bla U2OS receptor cells containing the human 5-HT_1A_R linked to a TEV protease site and a Gal4-VP16 transcription factor were tested for recruitment of β-arrestin. After thawing, the cells were cultured in the medium McCoy’s 5A with 10% FBS dialyzed, 0.1 mM NEAA, 25 mM HEPES, 1 mM sodium pyruvate, 100 μg/mL G-418, 100 U/mL penicillin/streptomycin Antibiotic, 200 μg/mL Zeocin, 50 μg/mL Hygromycin. In the experiment, cells were plated at 10 000 cells/well of a 384-well black, clear-bottom, tissue culture plate and grown for 12 h in an incubator (5% CO_2_, 37°C) in DMEM medium with 10% FBS added. The recruitment of β-arrestin were tested using the Tango LiveBLAzer assay kit according to the manufacturer’s instruction (Life Technologies). The assay plate was measured in FLUOstar Optima, a multifunction plate reader (BMG Lab Tech).

### Animals

The screening behavioral (FST, locomotor activity) and prolonged (the effect on metabolic parameters and weight gain as well as spontaneous locomotor and antidepressant-like activities) experiments were performed on male Swiss mice weighing 22–28 g (n = 230) and 20–22 g at the arrival (n = 30), respectively. The organ-pharmacological experiments were carried out on male guinea pigs (300–500 g, n = 2), while the effect of studied compounds on blood pressure was investigated in male Wistar rats (200–250 g, n = 24). The animals were purchased from the Animal House at the Faculty of Pharmacy, Jagiellonian University Medical College, Krakow, Poland (rats) or accredited Laboratory Animal Breeding Ilkowice, Słaboszów, Poland (mice and guinea pigs). The animals were kept in an environmental controlled room (temperature of 22 ± 2°C, humidity 55 ± 10%) on 12-h light/dark cycles (light on at 7:00 AM and off at 7:00 PM) and had free access to food (standard laboratory pellets) and tap water. The mice were housed in groups of 10 and rats and guinea pigs in groups of 4 in standard plastic cages of size (L × W × H) 382 × 220 × 150 mm, 378 × 217 × 180 mm and 590 × 380 × 230 mm, respectively. The following environmental enrichment materials were used: wooden blocks, paper tubs, paper stripes.

Pharmacokinetic study was conducted on male Swiss mice (20–25 g, n = 96) purchased from the Animal House at the Faculty of Pharmacy, Jagiellonian University Medical College, Krakow, Poland. During the habituation period, groups of 4 mice were kept in plastic cages (252 × 167 × 140 mm) in controlled environmental conditions described earlier and had free access to standard laboratory pellet and tap water.

All experiments were conducted in the light phase between 9 AM and 2 PM. Experimental groups consisted of 6–10 randomly selected animals, depending on the experiment (the detailed information is included in captions under each table and figure), and each animal was used only once. All surgery was performed under sodium pentobarbital (blood pressure measurement) or ketamine/xylazine anesthesia (pharmacokinetic studies), and all efforts were made to minimize suffering. Immediately after each experiment animals were sacrificed by trained staff using cervical dislocation (when blood or organs were collected) or carbon dioxide (after acute behavioral tests).

All the experimental procedures with mice, rats and guinea pigs were carried out in accordance with EU Directive 2010/63/EU and were approved by the Local Ethics Commission for Animal Experiments of Jagiellonian University in Krakow (Approval Nos.: 125/2017, 123/2015, 158/2017, and 127/2018). Moreover, in the studies, the 3R rule was applied in accordance with the relevant international and Polish regulations. In the event of a significant deterioration of animal health, we used early and humane endings of experimental procedures. Humane termination occurred when the animal had at least two symptoms such as convulsions, respiratory disturbance, movement disorder, immobility, lack of water and/or food intake, muscle relaxation, lack of touch response.

### Drug administration

The investigated compounds AZ-853 (8-(4-(4-(2-fluorophenyl)piperazin-1-yl)butyl)-1,3-dimethyl-1H-imidazo[2,1-f]purine-2,4(3H,8H)-dione and AZ-861 (1,3-dimethyl-8-(4-(4-(3-(trifluoromethyl)phenyl)piperazin-1-yl)butyl)-1H-imidazo[2,1-f]purine-2,4(3H,8H)-dione) were synthesised in the Department of Medicinal Chemistry, Faculty of Pharmacy, Jagiellonian University, and their synthesis was described earlier [8]. AZ-853 and AZ-861 were suspended in 1% aqueous solution of Tween 80, and WAY-100635 (Sigma, Germany) was dissolved in 0.9% saline. In acute experiments, the selected compounds AZ-853 (0.156–2.5 mg/kg) and AZ-861 (0.625–2.5 mg/kg) were injected *i*.*p*. once 60 min before tests, whereas WAY-100635 (0.3 mg/kg) was administered subcutaneously (*s*.*c*.) 45 min before the test. In interaction studies, the compounds were administered at doses for which the strongest anti-immobility effect in FST was observed, i.e. 1.25 mg/kg. In chronic experiments, AZ-853 and AZ-861 were administered *i*.*p*. once a day (between 9:00 AM and 10:00 AM) for 16 consecutive days at the minimum active doses selected from FST experiment, i.e. 0.625 and 1.25 mg/kg, respectively. All compounds were administered to mice at a volume of 10 ml/kg and to rats at a volume of 2 ml/kg. The control groups received 1% aqueous solution of Tween 80.

### Behavioral studies

#### Forced swim test

The experiment was carried out according to the method of Porsolt et al. [9]. Swiss albino mice were individually placed in a glass cylinder (25 cm high; 10 cm in diameter) containing 10 cm of water maintained at 23–25°C, and were left there for 6 min. The total duration of immobility was recorded during the last 4 min of a 6-min. A mouse was regarded as immobile when it remained floating on the water, making only small movements to keep its head above it.

#### Locomotor activity

Locomotor activity was recorded with an Opto M3 multi-channel activity monitor (MultiDevice Software v.1.3, Columbus Instruments). The Swiss albino mice were individually placed in plastic cages (220 **×** 120 **×** 130 mm) for 30 min habituation period, and then the crossings of each channel (ambulation) were counted from 3 to 6 min, that is the time equal to the observation period in FST. The cages were cleaned up with 70% ethanol after each mouse.

### LC/MS/MS analysis and pharmacokinetic studies

#### Sample pretreatment

Blood samples from the Swiss albino mice were collected at 0 (pre-dose), 5, 15, 30, 60, 120, 240 and 480 min after a compound administration. The blood samples and brain structures were collected under general anesthesia induced by *i*.*p*. injection of 50 mg/kg ketamine plus 8 mg/kg xylazine. The blood samples were taken into heparinized tubes, immediately centrifuged at 1000 × *g* for 10 min, and plasma was collected. The plasma samples and brain structures were immediately frozen at -30°C and -80°C, respectively.

The plasma and tissue sample pretreatment procedure involved acetonitrile precipitation. A 5 μL aliquot of the IS working solution (100 ng/mL) was added to 50 μL of the collected mouse plasma sample, which was then vortex-mixed for 10 seconds. Thereafter, 200 μL of acetonitrile was added, vortexed during 20 min, and then centrifuged (1000 × *g*, 10 min). The supernatant (100 μL) was then transferred to insert placed in an autosampler vial, and a 10 μL volume of this was injected onto the HPLC column. Tissue samples were thawed before use, and whole hippocampus, striatum and frontal cortex were weighted and placed in a glass mortar and pestle tissue grinder and homogenized with an appropriate amount of phosphate buffer (pH 7.4) in 1:5 ratio. Afterward, 50 μL of tissue homogenates were transferred to new Eppendorf tubes and spiked with 5 μL of the internal standard working solution. All samples were stored on ice during the preparation process. This was followed by procedures like those described above.

#### Materials and reagents

HPLC grade methanol, acetonitrile and reagent grade formic acid, hydrochloric acid, potassium dihydrogen phosphate, orthophosphoric acid and sodium chloride were purchased from Merck (Darmstadt, Germany)), xylazine were from Sigma-Aldrich (St. Louis, USA), ketamine was supplied by Vetoquinol (Gorzow Wielkopolski, Poland).

#### Instrumentation and operating conditions

The LC/ESI-MS/MS experiments were performed on an Applied Biosystems/MDS Sciex (Concord, Ontario, Canada) API 2000 triple quadrupole mass spectrometer equipped with an electrospray (ESI) ionization interface. This instrument was coupled to an Agilent 1100 (Agilent Technologies, Waldbronn, Germany) LC system. Data acquisition and processing were accomplished using Sciex Analyst 1.4.2 data collection and integration software.

#### Chromatographic conditions

An XBridge C18 (2.1 x 30 mm, 3.5 μm) analytical column (Waters, Milford, Massachusetts, USA) was used for compound separation. The temperatures of the column thermostat and the autosampler were set at 25°C and 10°C, respectively. The mobile phase consisted of a mixture of acetonitrile with addition of 0.1% formic acid (solvent A) and water with addition of 0.1% formic acid (solvent B) and was set at a flow rate of 0.6 mL/min. Starting amount of solvent A is 10%, isocratic elution from 0 to 3 min, and then gradient up to 90% of solvent A, and maintained to 10 min, and then gradient down to 10% of solvent A, and maintained to 15 min.

#### Mass spectrometric conditions

To find the optimal parameters of ion path and ion source for studied compounds the quantitative optimization was done by direct infusion of each compound at concentration of 1 μg/mL, and at a flow rate of 10 μL/min using a Hamilton syringe pump (Hamilton, Reno, Nevada).

The ion source parameters were as follows: ion spray voltage: 5500 V; nebulizer gas (gas 1): 20 psi; turbo gas (gas 2): 25 psi; temperature of the heated nebulizer: 400°C; curtain gas: 40 psi; collision gas: 6 psi. Mass spectra were acquired by SRM with precursor/predominant product ion transitions for each analyte. The mass spectral Q1→Q3 transitions monitored for AZ-853, AZ-861 and for IS (nebivolol), were m/z 453.9→235.1; 504.3→285.1 and 406.2→151.1, respectively. The peak widths of precursor and product ions were set to 0.7 full width half-height. Quantification was done *via* peak area ratio.

#### Pharmacokinetic parameters

Pharmacokinetic parameters were calculated by a non-compartmental approach from the average concentration values, using Phoenix WinNonlin software (Certara, Princeton, NJ 08540 USA). First order elimination rate constant (λ_z_) was calculated by linear regression of time versus log concentration. Next, the area under the mean serum and tissue concentration versus time curve (AUC_0→t_) was estimated using the log-linear trapezoidal rule (1), where C_i_ is the plasma concentration measured at t_1_.

$${AUC}_{0\to t}=\sum_{i=1}^{n}\left({(C}_{i}+C_{i+1})/2)∙(t_{i+1}-t_{i}\right).$$ (1)

Area under the first-moment curve (AUMC_0→t_) was estimated by calculation of the total area under the first-moment curve using the Eq (2), where C_i_ is the plasma concentration measured at t_1._

$${AUMC}_{0\to t}=\sum_{i=1}^{n}\left((t_{i}∙C_{i}+t_{i+1}∙C_{i+1})/2)∙(t_{i+1}-t_{i}\right).$$ (2)

Mean residence time (MRT) was calculated as (3):

$$\mathrm{MRT}=\frac{\mathrm{AUM}C_{0\to t}}{\mathrm{AU}C_{0\to t}}$$ (3)

Total clearance (Cl_T_) was calculated as (4):

$$Cl_{T}=\frac{F\cdot D_{i.p.}}{\mathrm{AU}C_{0\to t}}.$$ (4)

Volume of distribution (V_d_) was calculated as (5):

$$V_{d}=\frac{F\cdot D}{\lambda_{z}\cdot \mathrm{AU}C_{0\to t}},$$ (5)

where D_*i*.*p*._ is an *i*.*p*. dose of studied compounds.

### Safety profile studies

#### Blood pressure measurement

Male Wistar normotensive rats were anesthetized with thiopental (50–75 mg/kg, *i*.*p*.). The right carotid artery was cannulated with a polyethylene tube filled with heparin in saline to facilitate pressure measurement using the Datamax apparatus (Columbus Instruments, Columbus, Ohio, USA). The studied compounds were administered after a 15-min stabilization period.

In a separate series of experiments on anesthetized normotensive rats, the effect of studied compounds on the pressor response to methoxamine (Sigma Aldrich) (150 μg/kg) was investigated. Pressor responses to methoxamine injected intravenously were measured before and 30 min after the administration of the tested compound. The amplitudes of the pressure values were measured in 4 independent experiments.

#### Smooth muscle relaxant assay

The experiment was carried out according to the method described previously [10]. Male guinea pigs were sacrificed by cervical dislocation. The ileum was rapidly removed, rinsed, and cut into segments of 1.5–2.0 cm length. The tissues were mounted on a jacketed 20 mL organ bath (Tissue Organ Bath System; 750 TOBS, DMT, Denmark) filled with Krebs’s solution (NaCl 120 mM, KCl 5.6 mM, MgCl_2_ 2.2 mM, CaCl2 2.4 mM, NaHCO_3_ 19 mM, glucose 10 mM). The bath was aerated with 95 % O_2_/5 % CO2 and heated at 37°C. During the 60-min incubation period, the organ was rinsed 4 times with fresh Krebs’s buffer, and the tissues were also pre-stimulated by 60 mM KCl solution. The submaximal contraction induced by histamine (Sigma Aldrich) (2 μM) and carbachol (Sigma Aldrich) (100 μM) was evaluated in the presence and absence of the tested compounds. Amplitudes of contraction values were measured in 4 independent experiments.

#### Seventeen-day experimental procedure

**Fig 2:**
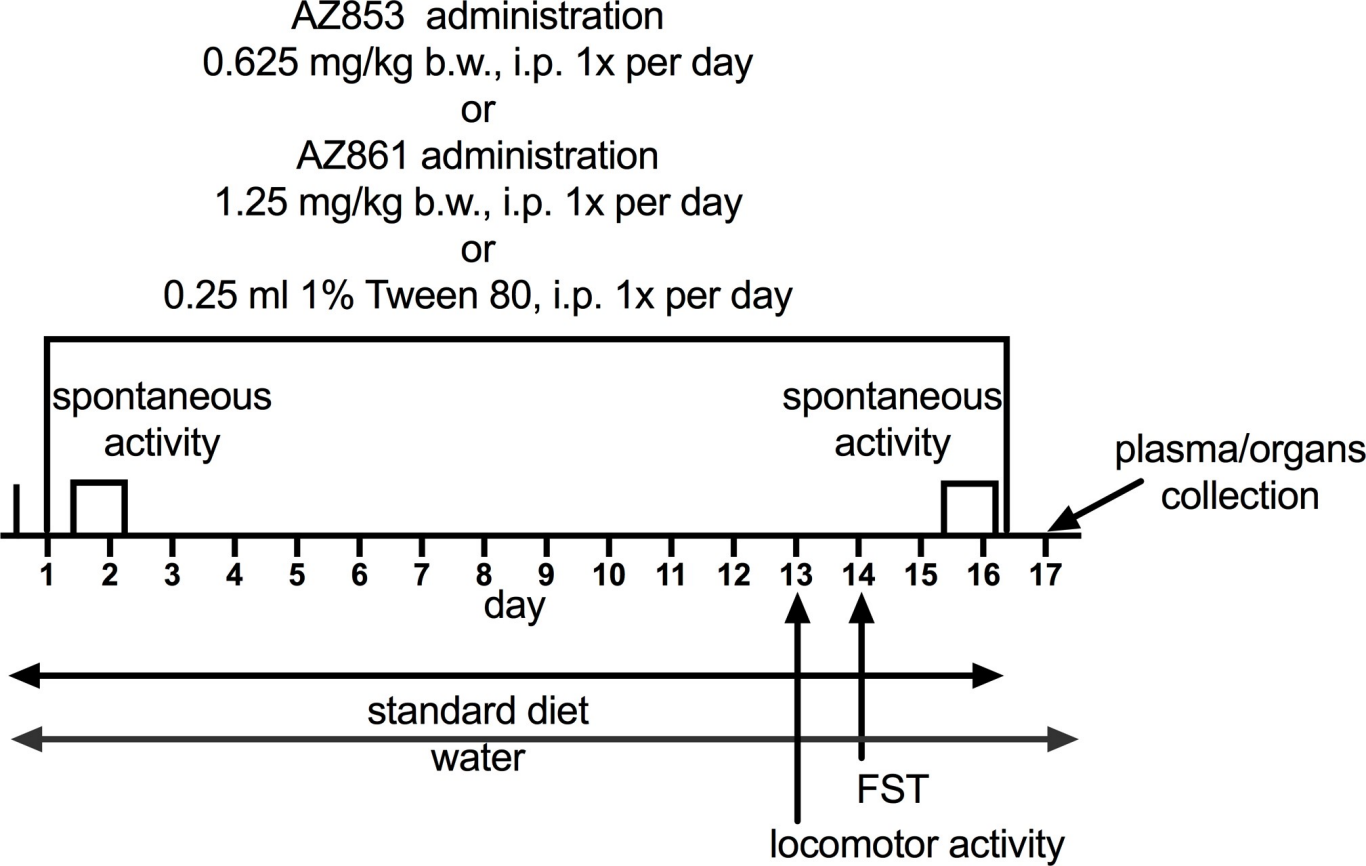
Scheme of seventeen-day experimental procedure.

Fig 2 shows the scheme of the 17-day experiment. The Swiss albino mice were used in the experiment. AZ-853 and AZ-861 were injected once daily for 16 consecutive days. On the 1st and 15th day the administration of the compounds, 18-h spontaneous animal activity was registered (see below). FST, as described earlier, was performed on the 14th day just before the next treatment. To ensure that the effects observed in FST are not the result of increased motility of animals, on the 13th day, just before the next administration, the locomotor activity was measured as described earlier, from 2 to 6 min after test start, i.e. in the period identical to the duration of FST. Blood and organ collection was performed on the 17th day at 24 h after the last dose and fasting.

#### Spontaneous activity monitoring

The spontaneous activity of the mice was measured on the 1st and 15th day of the treatment with a special, innovative radiofrequency identification system (RFID-system)—TraffiCage (TSE-Systems, Germany). The test was conducted according to the method described in the literature [11–13]. The animals had the transmitter (RFID) subcutaneously implanted, which tracked the time they spent in different areas of the cage. The data obtained were then grouped in a special computer program.

#### Body weight measurement and blood and organ collection

The body weight was measured daily immediately prior to administration of the drugs. On the 17th day, the blood was collected from the Swiss albino mice sacrificed by decapitation 20 min after *i*.*p*. administration of heparin (2500 units/mouse) and then centrifuged at 600 × *g* (15 min, 4°C) to separate plasma. The liver, heart, and kidney were also collected and weighed.

#### Biochemical analysis

Blood samples were collected from the Swiss albino mice 24 h after the last administration of the tested compounds or vehicle. To determine glucose, cholesterol and triglyceride levels in the plasma, standard enzymatic spectrophotometric tests (Biomaxima S.A. Lublin, Poland) were used. In the presence of appropriate enzyme, the substrate was converted to a colored compound. Absorbance was measured at a wavelength of 500 nm. To determine alanine aminotransferase (ALAT) or γ-glutamyl transpeptidase (γ-GTP) activities in the plasma, the standard enzymatic spectrophotometric test (Biomaxima S.A.) was used. Absorbance was measured at 340 nm (ALAT) or 405 nm (γ-GTP).

### Statistical analysis

The data of FST and locomotor activity were evaluated using one-way (when one drug was used) or two-way (when two drugs were administrated) analysis of variance (ANOVA) followed by Bonferroni’s post hoc test. Relaxation effects were expressed as percentage of inhibition of the maximal tension obtained with the contractile agent (histamine, carbachol or methoxamine). Their values and the results of biochemical studies (percent of tissue weight; glucose, triglyceride, and cholesterol levels; ALAT and γ-GTP activities) were compared using Student’s t test. Two-way variance analysis (ANOVA) followed by Bonferroni’s post-hoc test was used to evaluate changes in body weight, while the data of 18-h spontaneous activity were analysed using multiple t-test under the assumption that all rows were sampled from populations with the same scatter. All the results were expressed as means ± standard error of mean (SEM). A value of p<0.05 was considered statistically significant. Graph Pad Prism 6.0/7.0 and Statistica 13.1 were used for data analysis.

## Results

### Intrinsic activity of AZ-853 and AZ-861 at 5-HT_1A_R *in vitro* functional assays

The influence of the tested compounds on the inhibition of cAMP production, ERK1/2 phosphorylation and β-arrestin recruitment was determined. It was assumed that E_max_ values higher than 80% relative to the maximal effect of serotonin (5-HT) are a characteristic of a full agonist, values between 21% and 79% indicate a partial agonist, and E_max_ values of 20% or less are indicative of an antagonist.

The functional studies revealed that compounds AZ-853 and AZ-861 showed partial or full agonistic intrinsic activity in all tested assays (Table 1, S1 Fig). Compound AZ-853 partially inhibited cAMP formation with 2.3-fold lower efficacy (E_max_ = 43%) and 1.4-fold lower potency than 5-HT. In the same assay, AZ-861 showed 1.3-fold lower efficacy (E_max_ = 76%) but 2.6-fold higher potency than 5-HT.

**Table 1:** Intrinsic activity of AZ-853 and AZ-861 at 5-HT_1A_R *in vitro* functional assays.

5-HT_1A_ assay	Treatment	Agonist mode	
		E_max_ (%) ± SEM	pEC_50_ ± SEM
**cAMP**^a^	Serotonin	100±2.7	7.94±0.17
	WAY-100135	2±0.5^c^	n.c^c^
	AZ-853	43±0.5	7.80±0.41
	AZ-861	76±1.7	8.36±0.20
**pERK1/2**^a^	Serotonin	100±2.2	8.00±0.46
	WAY-100135	2±1.0^c^	n.c^c^
	AZ-853	60±2.0	8.18±0.33
	AZ-861	76±1.3	8.94±0.40
**β-arrestin**^b^	Serotonin	100±1.3	6.40±0.17
	WAY-100135	1±0.5^c^	n.c^c^
	AZ-853	93±1.7	8.38±0.01
	AZ-861	95±1.7	9.43±0.10

All the functional activity values were expressed as means from at least two experiments performed in duplicates.

E_max_−the maximum possible effect; pEC_50_ –the negative logarithm of concentration of compound where 50% of its maximal effect was observed; n.c.–not calculable. Results are expressed as percentage of maximal agonist response (serotonin 10^−6^ M).

^a^ The functional assay was performed using CHO-K1 cells

^b^ The functional assay was performed using U2OS cells (Tango LiveBLAzer assay kit)

^c^ Pytka et al. [14].

In the ERK1/2 phosphorylation (p-ERK1/2) assay, AZ-853 and AZ-861 showed partial agonistic properties by stimulating p-ERK1/2 with respectively 1.6-fold and 1.3-fold decreased efficacy (E_max_ = 60% and 76%), but with respectively 1.5-fold and 9.1-fold increased potency as compared to 5-HT.

The functional studies revealed that AZ-853 and AZ-861 activated β-arrestin recruitment with similar efficacy (E_max_ = 93% and 95%, respectively) as 5-HT (E_max_ = 100%), but their potency was increased 95-fold and 1000-fold as compared to that of the reference agonist.

To determine functional selectivity of the tested ligands, bias factors between particular pathways were calculated, according to the following Eq (6) [15,16]:

$$bias\;factor=log\left(\frac{{relative\;activity}_{12,lig}}{{relative\;activity}_{12,ref}}\right)=log[{\left(\frac{E_{{max}_{path1}}\times {EC}_{50-path2}}{{EC}_{50-path1}\times E_{{max}_{path2}}}\right)}_{lig}÷{\left(\frac{E_{{max}_{path1}}\times {EC}_{50-path2}}{{EC}_{50-path1}\times E_{{max}_{path2}}}\right)}_{ref}].$$ (6)

Bias factor ensures an equal comparison between all the pathways, considering the differences in E_max_ and EC_50_ values of both tested ligand and the reference compound 5-HT. Results for all the compounds are presented in Table 2. No significant differences were observed in the activation of signal transduction pathways by AZ-861 and AZ-863.

**Table 2:** Bias factors of compounds AZ-853 and AZ-861 and reference agonist serotonin at 5-HT_1A_R.

	5-HT_1A_R bias factor (logarithmic value)		
	ERK ½ vs. cAMP	ERK ½ vs. Β-arrestin	cAMP vs. β-arrestin
**Serotonin**	0.00	0.00	0.00
**AZ-853**	0.13	-0.08	-0.04
**AZ-861**	-0.03	-0.02	0.05

### Effects of acute administration of AZ-853 and AZ-861 on FST in mice

**Fig 3:**
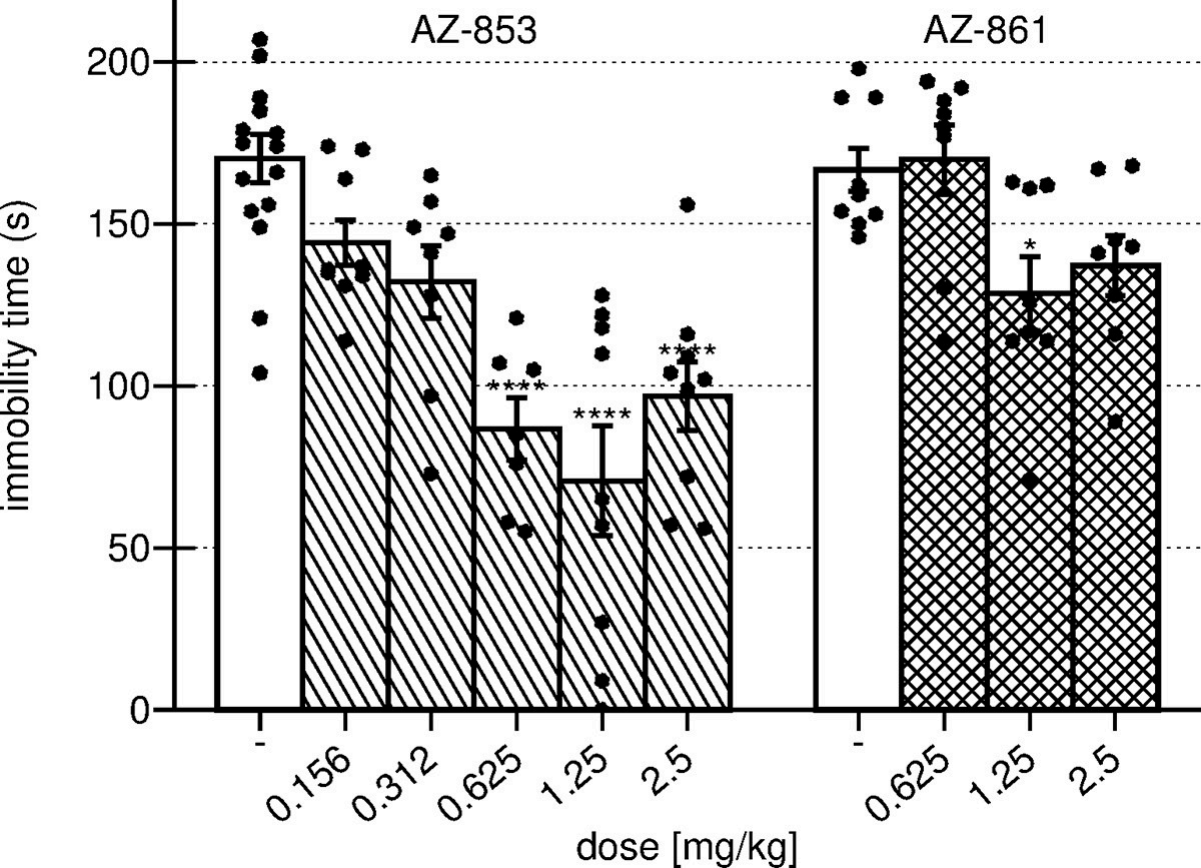
Effect of acute administration of AZ-853 and AZ-861 on the duration of immobility on FST in mice. Data represent mean ± SEM, n = 7–15 mice per group; one-way ANOVA followed by Bonferroni’s post hoc test; *p<0.05, ****p<0.0001 vs. respective control group.

The results presented in Fig 3 show that compound AZ-853 displayed strong and dose-dependent antidepressant-like activity by significantly reducing the immobility time of mice by approximately 50%, 58% and 45% at doses of 0.625, 1.25 and 2.5 mg/kg, respectively (F(5,52) = 14.822, p<0.0001). Compound AZ-861 significantly shortened the time of immobility by approximately 33% only at the middle dose of 1.25 mg/kg (F(3,29) = 4.7628, p<0.01).

To avoid biases related to the effect of treatment on animals’ motility that may affect the results obtained in FST, the spontaneous locomotor activity of mice was measured for 4 min, that is, the period identical to FST measurement. Both AZ-853 (0.625, 1.25, and 2.5 mg/kg) and AZ-861 (1.25 mg/kg) did not change the behavior of the animals (S1 Table, S1 Data).

### Effect of WAY-100635 administration on antidepressant-like activity of AZ-853 and AZ-861 on FST in mice

**Fig 4:**
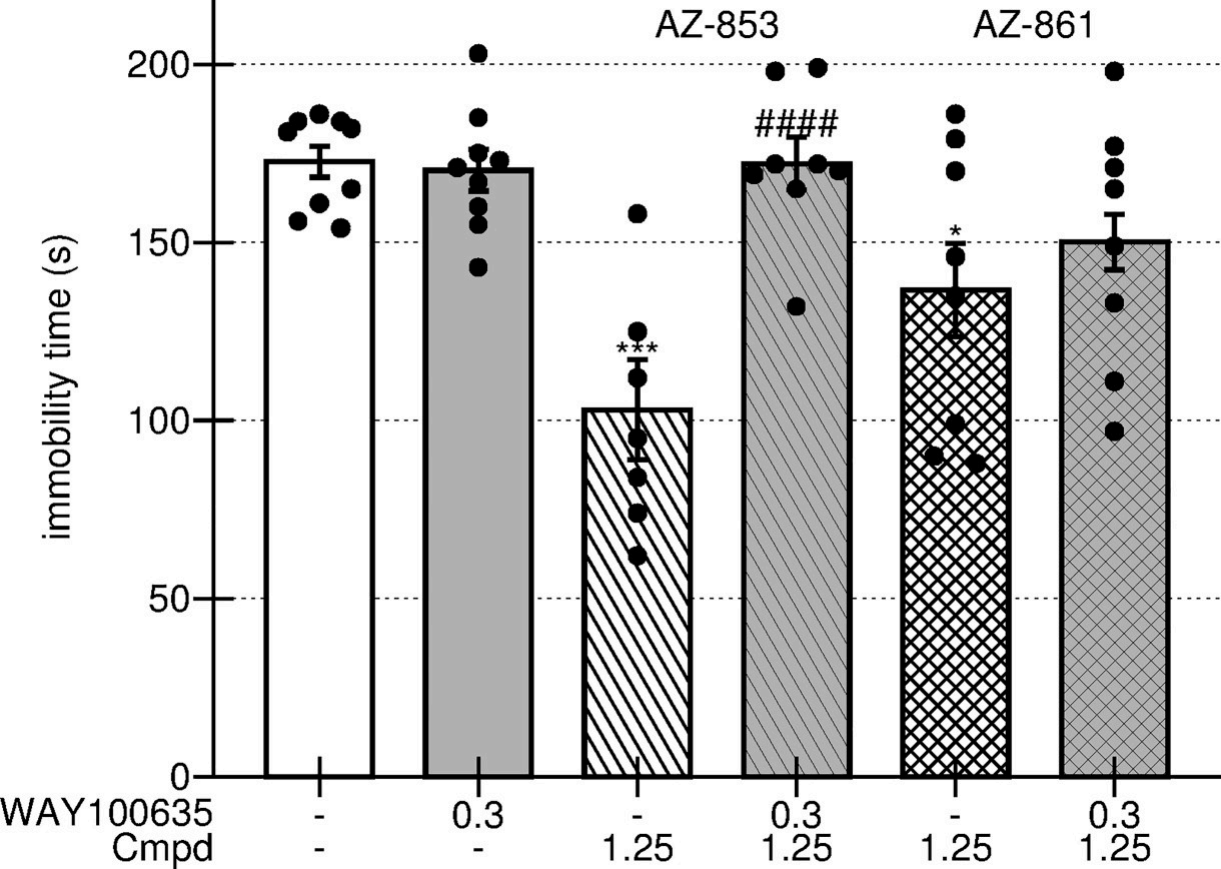
Effect of WAY-100635 administration on antidepressant-like activity of AZ-853 and AZ-861 on FST in mice. Data represent mean ± SEM, n = 7–9 mice per group; two-way ANOVA followed by Bonferroni’s post hoc test; *p<0.05, ***p<0.001 vs. control group, ####p<0.0001 vs. respective compound-treated group. Cmpd–the respective tested compound.

In interaction studies, the compounds were administered at doses for which the strongest anti-immobility effect in FST was observed. WAY-100635 administered alone at a dose of 0.3 mg/kg had no effect on immobility, but it completely abolished the antidepressant-like action of AZ-853 administered at a dose of 1.25 mg/kg (Fig 4). Two-way ANOVA results showed significant interaction (F(1,30) = 18.034, p<0.001). The concomitant administration of the active dose of AZ-861 (1.25 mg/kg) and WAY-100635 (0.3 mg/kg) led to attenuation of the anti-immobility effect of AZ-861, but two-way ANOVA results did not show statistically significant interaction (F(1,30) = 0.94270, ns) (Fig 4).

WAY-100635 (0.3 mg/kg) when given alone or combined with AZ-853 (1.25 mg/kg) or AZ-861 (1.25 mg/kg) did not change the locomotor activity of the animals (S1 Table, S1 Data).

### Effects of repeated administration of AZ-853 and AZ-861 on FST in mice

**Fig 5:**
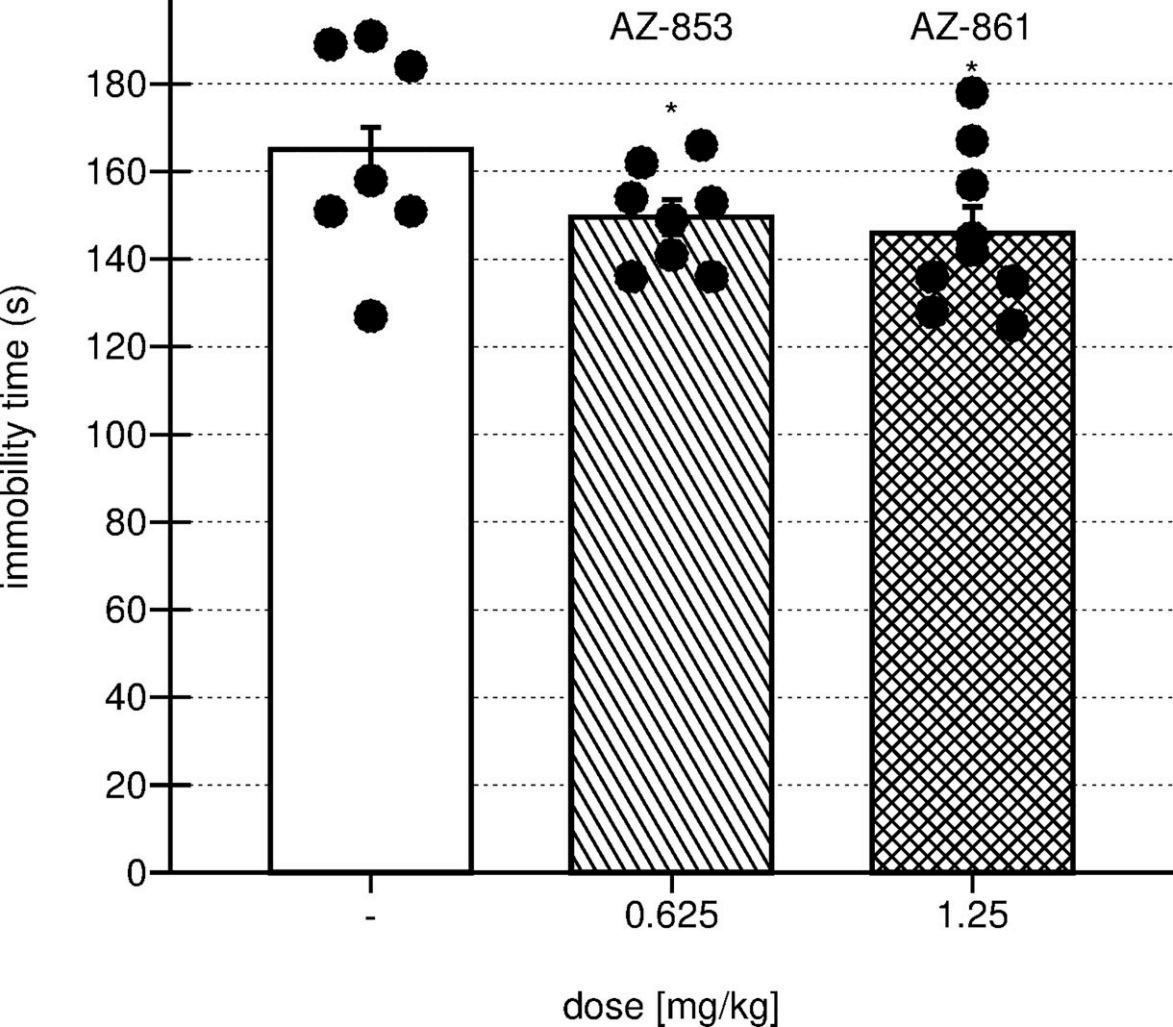
Effect of repeated administration of AZ-853 and AZ-861 on duration of immobility on FST in mice. AZ-853 and AZ-861 administered *i*.*p*. once daily for 13 consecutive days. Data represent mean ± SEM, n = 7–8 mice per group; one-way ANOVA followed by Bonferroni’s post hoc test; *p<0.05 vs. control group.

As shown in Fig 5, both AZ-853 (F(1,14) = 5.7973, p<0.05) and AZ-861 (F(1,15) = 5.8597, p<0.05) administered for 13 consecutive days at the minimum effective doses, i.e. 0.625 mg/kg and 1.25 mg/kg respectively, decreased the immobility time in FST in mice.

Prolonged administration of both compounds did not influence the motility of animals measured during the 4-min period (S2 Table, S2 Data).

### Effects of acute administration of AZ-853 and AZ-861 on pharmacokinetic parameters in mice

**Fig 6:**
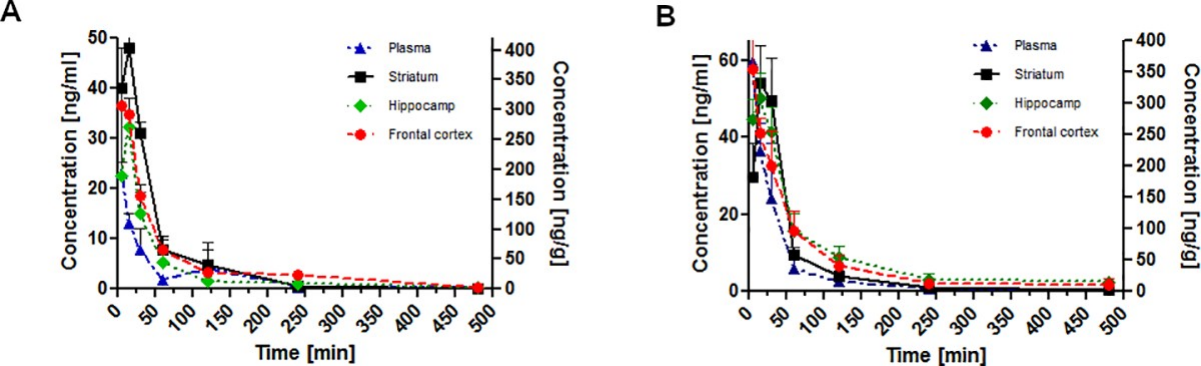
Concentration–time profiles of AZ-853 (A) and AZ-861 (B) in mouse plasma and different brain regions. AZ-853 administered *i*.*p*. at the dose of 0.625 mg/kg and AZ-861 administered *i*.*p*. at the dose of 1.25 mg/kg. Concentrations of the tested compounds in plasma and brain are expressed as ng/mL and ng/g, respectively. Data represent mean ± SEM, n = 6 mice per group per time point.

The plots of concentrations (mean ± SD) measured in plasma, hippocampus, striatum, and frontal cortex versus time profile for AZ-853 and AZ-861 after *i*.*p*. administration at the lowest antidepressant active dose of 0.625 mg/kg are shown in Fig 6(A). Similar linear plots for AZ-861 administered *i*.*p*. at the lowest antidepressant active dose of 1.25 mg/kg is shown in Fig 6(B) (see also S3 Data).

The pharmacokinetic parameters calculated by the non-compartmental approach are given in Tables 3 and 4.

**Table 3:** Pharmacokinetic parameters and brain structure uptake for AZ-853.

Parameter	Plasma	Hippocampus	Striatum	Frontal cortex
**AUC**_**0→t**_ **[ng · min/mL]**	1063	12618	21142	19232
**t**_**0.5**_ **[min]**	81.6	140.8	74.3	73.8
**MRT [min]**	85.8	71.6	56.3	90
**C**_**max**_ **[ng/mL]**	23.1	271.8	403.6	306.3
**t**_**max**_ **[min]**	5	15	15	5
**V**_**d**_**/F [L/kg]**	66.9	-	-	-
**Cl/F [L/min/kg]**	0.57	-	-	-
**Brain structure-to-plasma concentration ratio**	-	11.9	19.9	18.1

AZ-853 administered to mice *i*.*p*. at the dose of 0.625 mg/kg. AUC_0→t_−area under the curve from zero to last sampling time; t_0.5_ –terminal half-life; C_max_−maximum plasma concentration; t_max_−time to reach C_max;_ V_d_/F–apparent volume of distribution; Cl/F–apparent clearance; MRT–mean residence time.

**Table 4:** Pharmacokinetic parameters and brain structure uptake for AZ-861.

Parameter	Plasma	Hippocampus	Striatum	Frontal cortex
**AUC**_**0→t**_ **[ng · min/mL]**	2070	26132	18566	21790
**t**_**0.5**_ **[min]**	97.5	110.4	83	91.6
**MRT [min]**	52.3	107	56.9	92.3
**C**_**max**_ **[ng/mL]**	59.6	308.5	333.1	355
**t**_**max**_ **[min]**	5	15	15	5
**V**_**d**_**/F [L/kg]**	83.4	-	-	-
**Cl/F [L/min/kg]**	0.59	-	-	-
**Brain structure-to-plasma concentration ratio**	-	12.6	8.97	10.5

AZ-861 administered to mice *i*.*p*. at the dose of 1.25 mg/kg. AUC_0→t_−area under the curve from zero to last sampling time; t_0.5_ –terminal half-life; C_max_−maximum plasma concentration; t_max_−time to reach C_max;_ V_d_/F–apparent volume of distribution; Cl/F–apparent clearance; MRT–mean residence time.

After *i*.*p*. administration, the tested compounds rapidly, within 5 min (t_max_) achieved the maximum concentration (C_max_) in the blood. The lowest effective dose for AZ-861 was two times higher than for AZ-853, thus the C_max_ for AZ-853 (23.125 ± 15.99 ng/mL) was *ca*. 2 times lower than C_max_ for AZ-861 (59.6 ± 38.6 ng/mL). Both AZ-853 and AZ-861 were eliminated relatively rapidly from mouse body with t_0.5_ *ca*. 1.5 h. For AZ-853 and AZ-861, the volume of distribution values were 67 L/kg and 83 L/kg, respectively. Very high volume of distribution (above 1 L/kg) indicates the ability of compounds to bind to and to accumulate in tissues. Using the area under the curve (AUC) values for AZ-853 and AZ-861 (Fig 6), the ratios between the AUC values in hippocampus, striatum, frontal cortex and in plasma were calculated. Consequently, the compounds were characterized by a very high permeability of the brain-blood barrier and exhibited significant distribution in brain structures from 5 to 15 min (Tables 3 and 4). Compound AZ-853 showed a greater extent of penetration into brain structures than AZ-861. For AZ-853, the ratios of hippocampus/plasma (H/P), striatum/plasma (S/P), and frontal cortex/plasma (FC/P) were 11.9, 19.9, and 18.1, respectively. For AZ-861, the ratios of H/P, S/P, and FC/P were 12.6, 8.97, and 10.5, respectively.

### Effects of acute administration of AZ-853 and AZ-861 on blood pressure in normotensive rats

**Fig 7:**
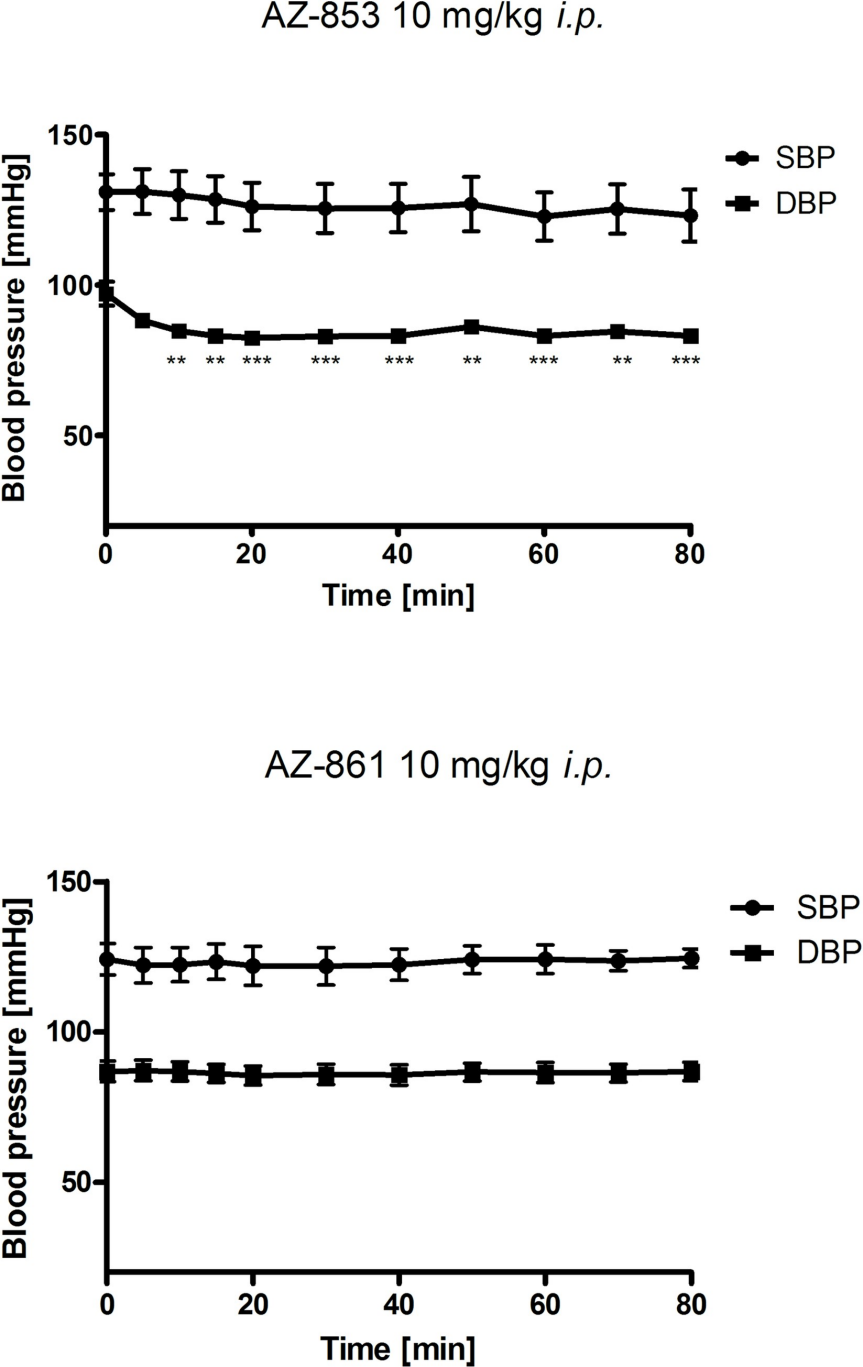
Effects of AZ-853 and AZ-861 on systolic and diastolic blood pressure after *i*.*p*. administration to normotensive anesthetized rats. All values represent the mean ± SEM, n = 6 rats per group. Statistical analysis was performed using one-way ANOVA followed by Bonferroni’s post hoc test; **p<0.01, ***p<0.001 compared to the time zero. SBP–systolic blood pressure, DBP–diastolic blood pressure.

Compound AZ-853 alone administered at a dose of 10 mg/kg significantly reduced diastolic blood pressure by 13–15% in relation to the baseline value. However, it did not significantly affect the systolic blood pressure. Compound AZ-861 administered at the identical dose did not significantly affect the arterial pressure of normotensive rats (Fig 7, S4 Data)

### Adrenolytic activity of acute administration of AZ-853 and AZ-861 in rats

**Fig 8:**
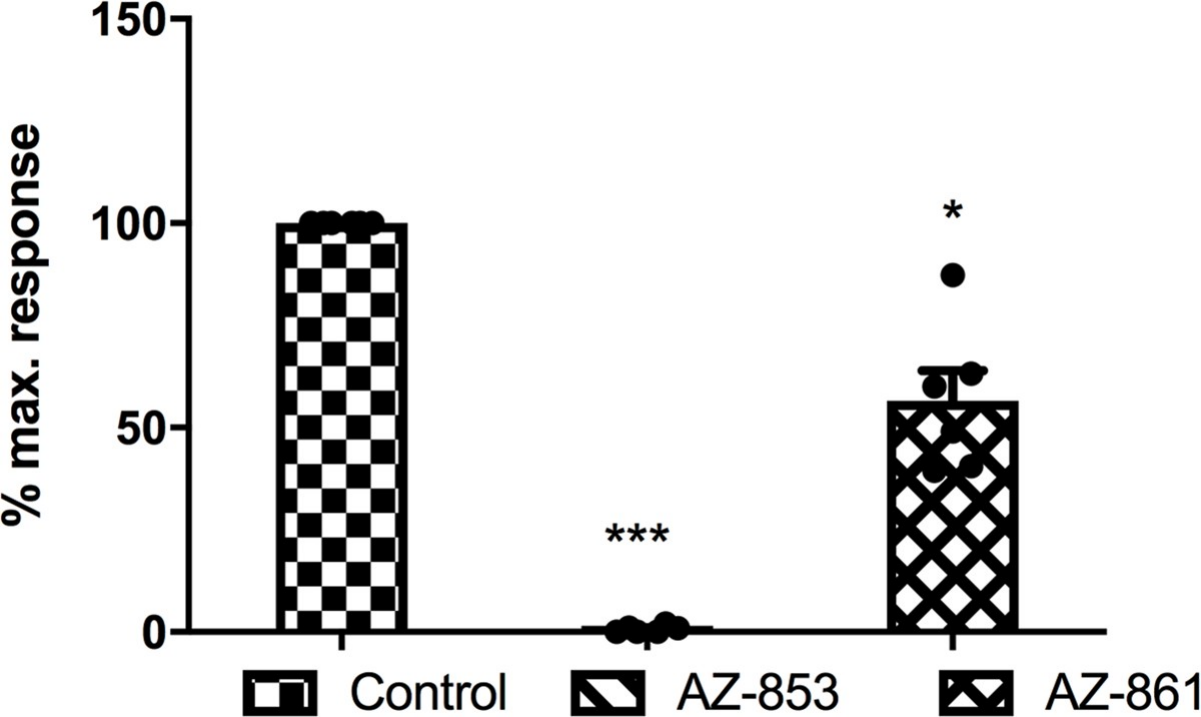
Effects of AZ-853 and AZ-861 on the blood pressure response to methoxamine (150 μg/kg). All values represent the mean ± SEM, n = 6. Statistical analysis was performed using Student’s t-test; *p<0.05, ***p<0.001 vs. control group.

Administration of AZ-853 at a dose of 10 mg/kg 30 min prior to methoxamine (150 μg/kg) resulted in a mean reduction of blood pressure in rats by 95.5 mmHg in comparison to values observed after the administration of methoxamine alone as a control. Administration of AZ-861 (10 mg/kg) induced the mean reduction of blood pressure in rats by 40.45 mmHg when compared to values after the administration of methoxamine alone as a control (Fig 8).

### Anticholinergic and antihistaminergic activities of AZ-853 and AZ-861 *in vitro*

**Fig 9:**
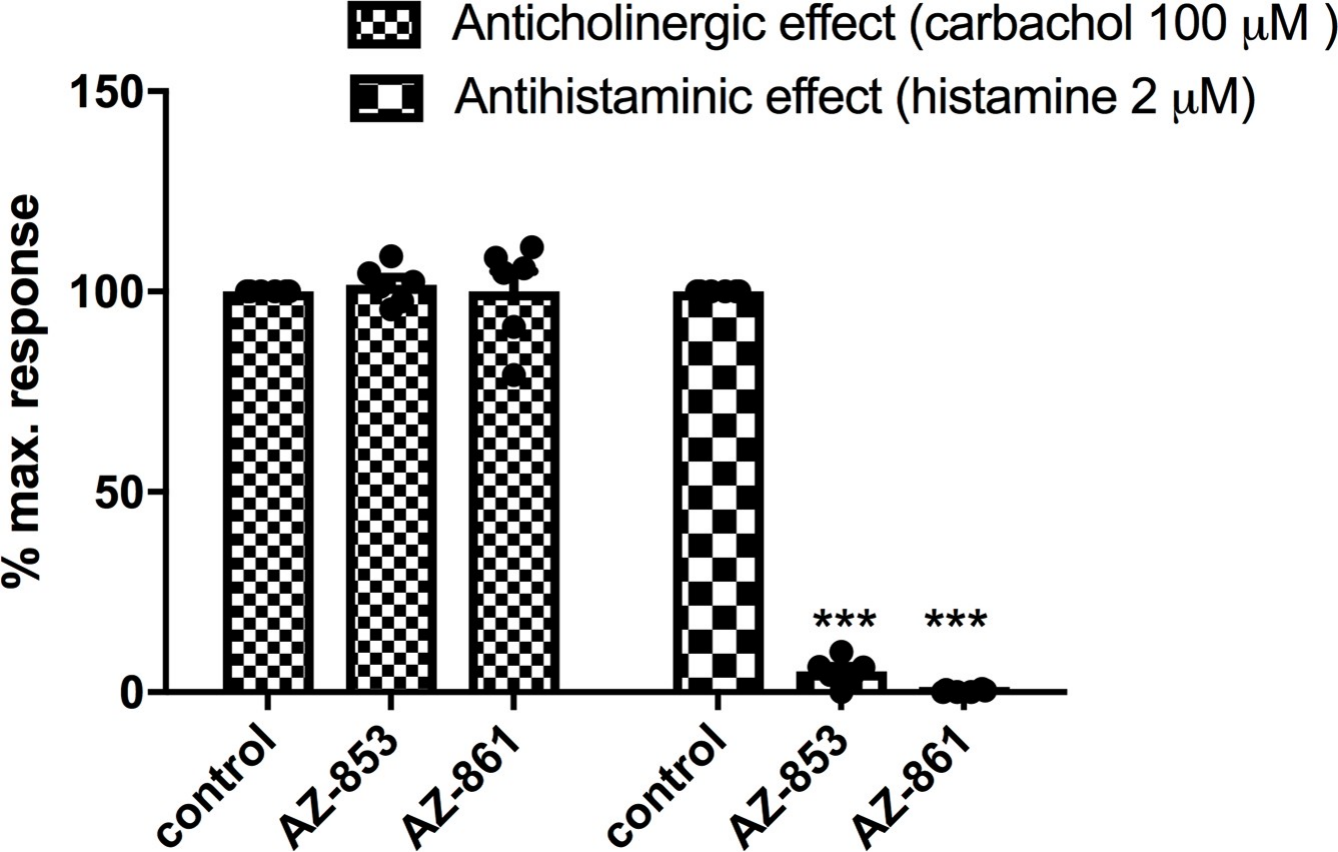
Effects of AZ-853 and AZ-861 on carbachol and histamine-induced smooth muscle contraction in isolated small intestine of guinea pig. All values represent the mean ± SEM, n = 6. Statistical analysis was performed using Student’s t-test; ***p<0.001 vs. control.

After the administration of AZ-853 and AZ-861 at the concentration of 1 μM, no inhibition of carbachol-induced contraction of the small intestine was observed. For AZ-853 and AZ-861, the contraction inhibition was on average 2.5% and 0.17%, respectively. The contraction caused by carbachol as the control sample was accepted as 100% (Fig 9).

AZ-853 and AZ-861, administered at the concentration of 1 μM, almost completely inhibited histamine-induced contraction of the isolated small intestine of guinea pig. In both cases, the inhibition reached almost 100%, i.e. 94.85% for AZ-853 and 99.79% for AZ-861. The contractions caused by histamine as the positive control were accepted as 100% (Fig 9).

### Effects of repeated administration of AZ-853 and AZ-861 on body weight of mice

**Fig 10:**
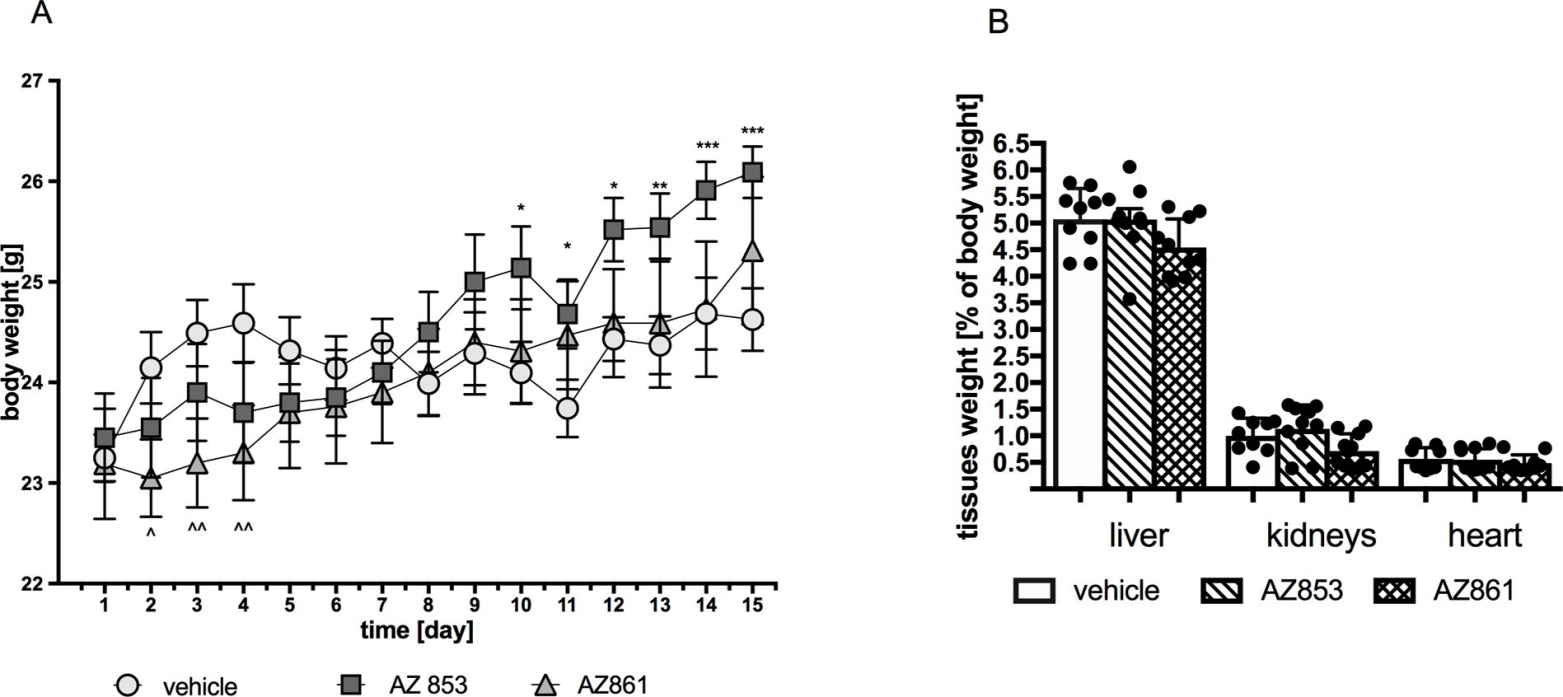
Effects of AZ-853 and AZ-861 on body (A) and tissues weight (B) of mice. Panel (A): data represent mean ± SEM, n = 10 per group. Statistical analysis was performed using two-way ANOVA followed by Bonferroni’s post-hoc test. Panel (B) data represent mean ± SEM, n = 9–10 per group. Statistical analysis was performed using Student’s t-test. *p<0.05, **p<0.01, ***p<0.001 vs. control group.

A slight but significant increase in body weight was observed in animals treated with AZ-853 at the dose of 0.625 mg/kg from the 10th day of administration. The results are shown in Fig 10A. In animals treated with AZ-861 at the dose of 1.25 mg/kg, a slight and significant decrease in body weight was observed on the 2nd, 3rd, and 4th day of administration. In the following days, no significant differences in body weight were observed as compared to those of the control animals. The results are shown in Fig 10A and in S5 Data.

### Effects of repeated administration of AZ-853 and AZ-861 on mouse liver, kidney, and heart weight

As shown in Fig 10B, the repeated administration of neither AZ-853 (0.625 mg/kg) nor AZ-861 (1.25 mg/kg) influenced liver, kidney, and heart weight expressed as the percentage of total body weight when compared with those measured in the control animals.

### Effects of repeated administration of AZ-853 and AZ-861 on plasma glucose, triglyceride, and cholesterol concentrations in mice

**Fig 11:**
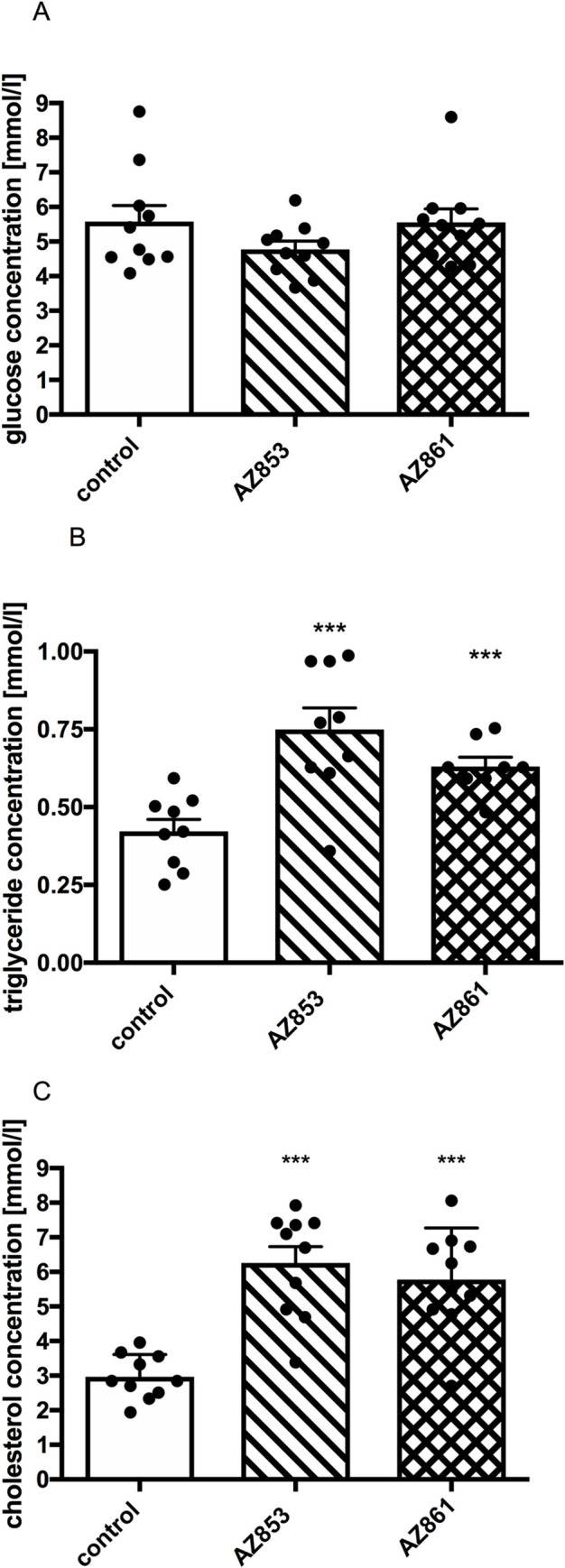
Effects of repeated administration of AZ-853 and AZ-861 on glucose, triglyceride, and cholesterol concentration in mouse plasma. Data represent mean ± SEM, n = 9–10 per group. Statistical analysis was performed using Student’s t-test. *p<0.05, **p<0.01, ***p<0.001 vs. control.

In the group of mice administered AZ-853 at the dose of 0.625 mg/kg b.w./day, the level of triglycerides and total cholesterol increased significantly as compared to those measured in the control animals. Similarly, after the administration of AZ-861 at the dose of 1.25 mg/kg b.w./day, the level of triglycerides and total cholesterol increased significantly as compared to those in the control animals. No differences were observed in plasma glucose levels between the groups. The results are presented in Fig 11.

### Effects of repeated administration of AZ-853 and AZ-861 on plasma ALAT and γ-GTP activity

**Fig 12:**
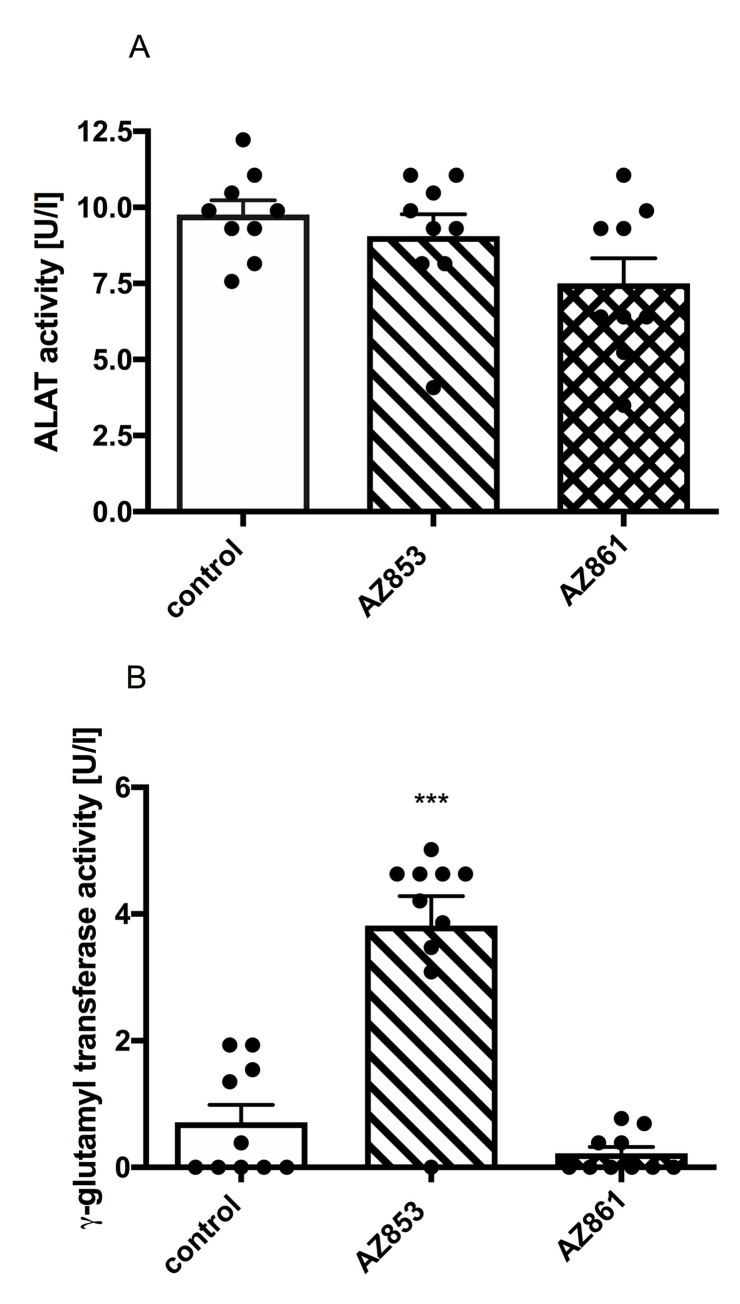
Effects of repeated administration of AZ-853 and AZ-861 on the activity of ALAT (A) and γ-GTP (B) in mouse plasma. Data represent mean ± SEM, n = 9–10 per group. Statistical analysis was performed using Student’s t-test; ***p<0.001 vs. control.

The administration of AZ-853 and AZ-861 at the dose of 0.625 and 1.25 mg/kg b.w./day, respectively, did not lead to significant changes in ALAT activity measured in mouse plasma (Fig 12A).

AZ-853 significantly increased γ-GTP activity by approximately 435% while AZ-861 did not change activity of this enzyme measured in mouse plasma. The results are shown in Fig 12B).

### Effects of acute and repeated administration of AZ-853 and AZ-861 on the spontaneous activity of mice

**Fig 13:**
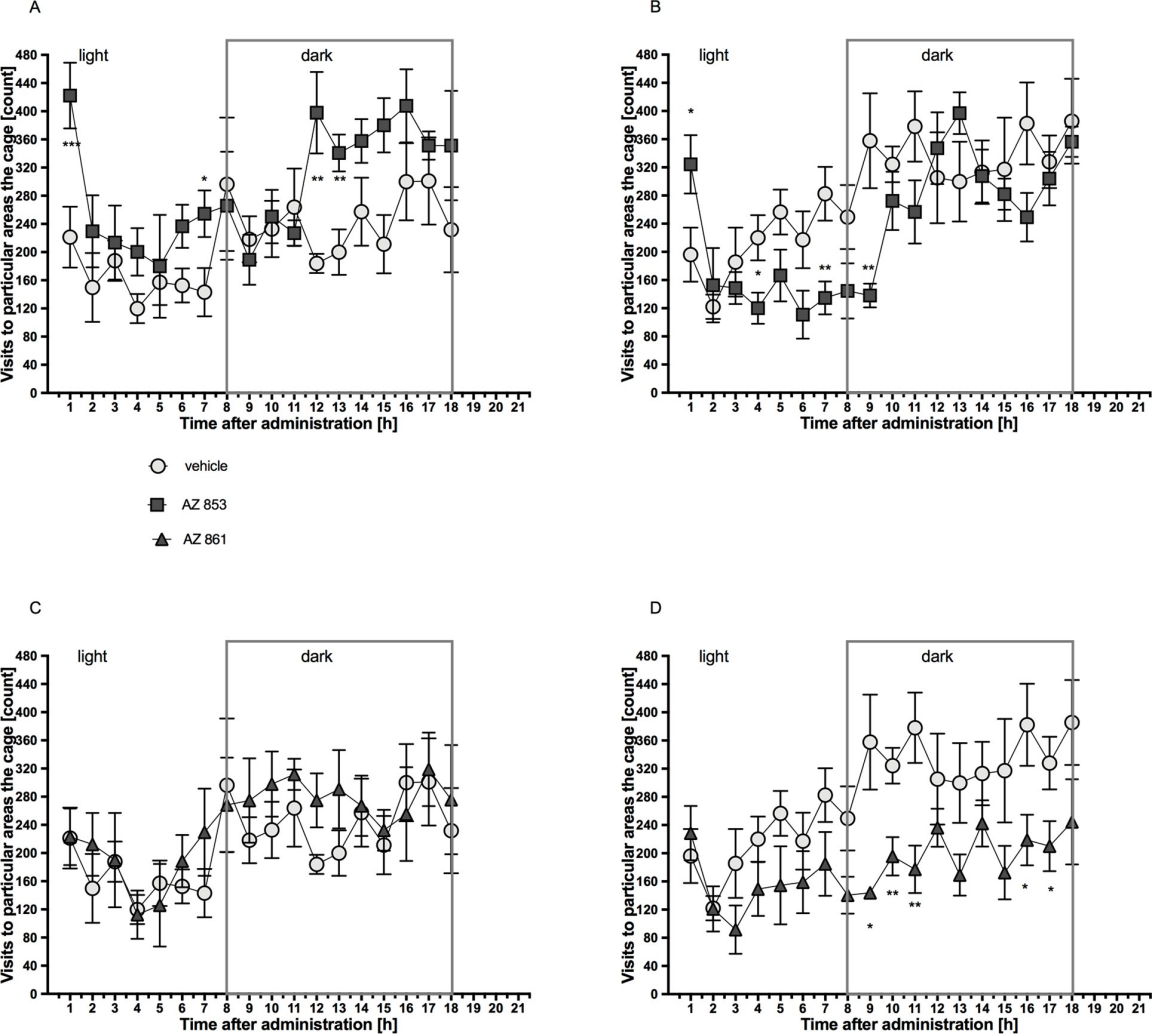
Effects of AZ-853 and AZ-861 administration on the spontaneous activity of mice during light and dark phases. A–after the 1st administration of AZ-853, B–after the 15th administration of AZ-853, C–after the 1st administration of AZ-861, D–after the 15th administration of AZ-861. Each plot represents mean ± SEM for n = 7–8 per group. Statistical analysis was performed using multi-t test, *p<0.05, **p<0.01 vs. control.

Mice treated with AZ-853 at the dose of 0.625 mg/kg b.w./day had increased spontaneous activity in the 1st, 7th, 12th and 13th hour after administration as compared to the control mice. After the 15th administration, a significant increase in activity was observed only in the 1st hour, but in the 4th, 7th and 9th hour after administration, a significant reduction in spontaneous activity was observed as compared to that in control mice (Fig 13A and 13B, S6 Data). No effect on locomotor activity was observed after single administration of AZ-861 at the dose of 1.25 mg/kg b.w./day. In contrast, after a 15th administration, a significant decrease in spontaneous activity was observed as compared to that in the control mice at 9th, 10th, 11th, 16th, and 17th hour after administration (Fig 13C and 13D, S6 Data).

## Discussion

AZ-853 and AZ-861 are chemical analogs that differ only in the quality of the fluorine substituent in the phenyl ring attached to piperazine. A small change in the chemical structure may cause differences not only in chemical and physical properties but also in pharmacological and pharmacokinetic characteristics (e.g., paliperidone vs. risperidone) [17–20]. Our previous investigations revealed that compounds AZ-853 and AZ-861 possess high affinity for serotonergic 5-HT_1A_R and are 5-HT_1A_R antagonists in CHO-K1 cells in the Ca^2+^ mobilization assay, with AZ-861 showing 2-fold higher K_b_ value [8]. Like other G protein-coupled receptors (GPCRs), 5-HT_1A_R binds to a wide variety of second messengers, i.e., adenyl cyclase, protein kinase A, phospholipase C, nitric oxygen synthase, and Ca^2+^ [21]. Over the past 20 years, several studies have demonstrated that intracellular signaling cascades may be activated with different preferences. GPCR ligands may stabilize different active receptor conformations, leading to the activation of distinct signaling pathways or they could even have opposite efficacies toward different signaling pathways. Such dual efficacies were reported for ligands that acted on adrenergic β_1_ and β_2_ (propranolol [22]), histamine H_3_ (proxyfan [23]), opioid δ (ICI174864 [24]), and serotonin 5-HT_1A_ (HBK-17 [14]) and 5-HT_2C_ receptors (quipazine [25]). The ability of a ligand to direct a G protein-coupled receptor toward a conformation that prefers one signaling pathway over another (functional selectivity or biased agonism) offers new opportunities for developing pathway-selective drugs; this might result in novel treatment strategies, i.e., pharmacological activity without inducing unwanted effects. In the present study we broadened the *in vitro* activity of AZ-853 and AZ-861 and showed that both the compounds exhibited partial agonistic properties in two functional assays with 5-HT_1A_R, i.e., inhibition of cAMP production and ERK1/2 phosphorylation as well as agonist activity in β-arrestin recruitment. In the latter, the efficacy of AZ-853 (E_max_ = 93%) and AZ-861 (E_max_ = 95%) was similar relative to that induced by the endogenous agonist (E_max_ = 100%), and their potency (pEC_50_) was even higher (96-fold and 1000-fold, respectively) than that of 5-HT. The obtained findings agree with previously published data. For 5-HT_1A_R ligands, biased agonism occurs, and the same molecule can show different effects on signal transduction pathways associated with one receptor depending on its localization in the respective brain structures [26]. Hence, although both compounds were initially defined as 5-HT_1A_ antagonists [8], on the basis of more extensive research, they should be classified as 5-HT_1A_R partial agonists, as they act differently depending on the brain structure. Despite having much higher potency than that of 5-HT, the calculated 5-HT_1A_R bias factor indicates that both the compounds did not preferentially activate any intracellular pathway and thus did not show functional selectivity relative to the endogenous agonist.

5-HT_1A_R is known to mediate antidepressant activity in rodents and humans. It has been repeatedly shown that 5-HT_1A_R agonists, partial agonists, and antagonists exhibit or accelerate antidepressant-like effects in animal behavioral tests [for review see [27]]. Some ADDs having 5-HT_1A_R agonistic properties and functioning as 5-HT transporter inhibitors (vilazodone or vortioxetine) are used clinically [28,29]. The anxiolytic drug buspirone (5-HT_1A_R agonist) is used as an augmentation strategy in MDD [30]. Likewise, pindolol, a 5-HT_1A_R /β-adrenoceptor antagonist, accelerated the onset of action and enhanced the antidepressant effect of SSRIs in adult patients with depressive disorders [31]. Thus, it is not surprising that 5-HT_1A_R continues to receive much interest as the target of antidepressant therapy [for review see [32]].

In addition to affinity for 5-HT_1A_R, AZ-853 and AZ-861 bind with moderate (AZ-861) or low (AZ-853) potency to 5-HT_7_R [8]. 5-HT_7_R is mostly located in brain structures involved in affective processes (i.e., hippocampus, pre-limbic cortices) [33]. 5-HT_7_R antagonists produced a significant antidepressant effect and enhanced the antidepressant activity of citalopram (SSRI) in pre-clinical studies in rodents [34,35]. Thus, 5-HT_7_R antagonists seem to be promising components of antidepressant therapy. It is worth noting that vortioxetine is a 5-HT_7_R antagonist [29], and some atypical antipsychotics, namely amisulpride, lurasidone and aripiprazole, show a clinically established antidepressant activity that might be mediated by 5-HT_7_R antagonism [36–38].

Considering the above-mentioned data, we performed FST in mice for the preliminary evaluation of antidepressant-like properties of compounds AZ-853 and AZ-861. FST is a widely used, well-established animal experiment tool for predicting the therapeutic potential of novel bioactive substances. We demonstrated that AZ-853 and AZ-861 showed antidepressant-like activity by causing a significant decrease in immobility time of mice. The effect of acute administration of AZ-861 revealed that only at one dose of 1.25 mg/kg, the compound produced a U-shaped dose–response relationship and was almost 2-fold weaker than AZ-853 at doses of 0.625–2.5 mg/kg. The antidepressant-like activity of both compounds was maintained after repeated 13-day application; however, it was less pronounced than that observed after acute administration but still significant. Because changes in the immobility time could also result from the effects on locomotor activity caused by central nervous system (CNS) stimulants, it was confirmed that active doses of both compounds did not increase mouse locomotor activity, which was measured over a period identical to the duration of FST. Thus, the present study provides pharmacological evidence for the specific antidepressant-like effects of AZ-853 and AZ-861.

Bearing in mind that depression is a complex disorder of psychological, neuroendocrine, and somatic symptoms, which are impossible to reproduce in animal models, we are aware that the use of only one test to fully characterize antidepressant properties of compounds has limitations. First, FST is not a full-spectrum analog of human depression, and it assesses only one aspect of depressed mood, i.e., despair-like behavior, which is thought to correspond to disengagement in depressive patients [39]. Moreover, it has been suggested that immobility could be interpreted as an adaptive behavior that allows an animal to survive in adverse conditions without unnecessary energy expenditure [40]. Some concerns are also raised regarding the effectiveness of acute treatment using new compounds/drugs in FST, while in clinic, chronic use is necessary to reveal the antidepressant effects of ADDs [41–43]. Thus, a thorough evaluation of antidepressant properties of new compounds requires the use of a battery of tests that assess other symptomatic dimensions of depressive states. However, these limitations should not devaluate the usefulness of FST as a drug discovery tool. It should be emphasized that FST detects antidepressant-like activity of the majority of clinically used ADDs and differentiates ADDs from anxiolytic and antipsychotic drugs. Therefore, it seems to be a suitable screening test in the preliminary search for novel compounds with potential antidepressant activity [43].

To verify whether 5-HT_1A_R stimulation is responsible for the activity of the tested compounds in FST, we performed the test again by using a selective 5-HT_1A_R antagonist (WAY-100635). We observed that WAY-100635 completely abolished the antidepressant-like effect of AZ-853, but only attenuated the effect of AZ-861. This implies that the antidepressant-like activity of AZ-853 depended on its 5-HT_1A_R agonistic properties. Instead, the anti-immobility action of AZ-861 is only partially determined by its 5-HT_1A_R agonist characteristics, and other mechanisms, including 5-HT_7_R antagonism, may be responsible for its antidepressant-like effect. The U-shape antidepressant action of AZ-861 is similar to the anti-immobility effect of SB-269970, a selective 5-HT_7_R antagonist [34], and this compound has 5-fold higher affinity for 5-HT_7_R than AZ-853.

At this stage of experimentation, it is difficult to explain which 5-HT_1A_R-coupled signal transduction pathway is involved in the antidepressant-like activity of the studied compounds. It can be speculated that the activation of p-ERK1/2 may have some relevance. There is increasing evidence implicating the role of ERK1/2 in the regulation of depressive behavior in both humans and animals. The suppression of the ERK signaling pathway was shown in animal models of depression [44] and in post-mortem prefrontal cortex and hippocampus of depressed suicide victims [45,46]. It was also demonstrated that the blockade of MEK/ERK signaling induced depression-like behavior in mice and blocked or reduced the antidepressant-like effects of desipramine and sertraline in FST [47], and that imipramine averted the disruption in MEK/ERK signaling induced by restraint stress in mice [44]. It is worth mentioning that ß-arrestins not only desensitize G-protein-dependent signal pathways but also promote novel pathways of signal transduction, e.g., ERK, JNK, p38, or Akt (for review see [48]). Thus, it is possible that the observed increase in ERK1/2 phosphorylation might also be due to the activation of ß-arrestin recruitment [49].

Phosphodiesterases (PDEs) have been shown to play distinct roles in the processes of emotion, while selective PDE inhibitors can modulate mood and emotional states by preventing the breakdown of cAMP and/or cGMP. Some members of the PDE family, such as PDE4B and PDE10A, may be good drug targets because of their presence in areas of the brain associated with the site of action of psychotropic drugs, and their inhibitors therefore have potential therapeutic relevance [50]. Certainly, we could exclude the inhibition of PDE4B and PDE10A as a potential mechanism of antidepressant-like action of AZ-853 and AZ-861; this is because in a previous *in vitro* study using a bioluminescent detection system, we demonstrated that both compounds did not induce PDE4B and PDE10A inhibitory activity [8].

The lipophilicity of a drug candidate can affect its pharmacodynamic and pharmacokinetic properties. In particular, the ability of a molecule to cross the cell membrane depends on its partition coefficient (log P), which is still the generally accepted and primarily applied descriptor of lipophilicity while searching for new drug candidates. The so-called “rule of five” defines the range of log P for good intestinal permeability and penetration to the brain as log p < 5. The estimated log p values for AZ-853 and AZ-861 were almost within the limits of “rule of five”, i.e., 5.34 and 5.75, respectively [8], which has been confirmed in pharmacokinetic experiments. We studied the pharmacokinetic profile of both the tested compounds administered *i*.*p*. at minimal antidepressant doses, i.e., 0.625 mg/kg (AZ-853) and 1.25 mg/kg (AZ-861). Both AZ-853 and AZ-861 achieved peak concentration in the blood within 5 min of administration, and their half-life in plasma was 81 and 97 min, respectively. The very high volume of distribution (over 67 L/kg and 84 L/kg for AZ-853 and AZ-861, respectively) indicates that the tested compounds bind to and accumulate in tissues. AZ-853 and AZ-861 showed high penetration into brain structures (hippocampus, striatum, and frontal cortex). Compound AZ-853 reached higher concentrations and remained longer in the studied brain areas than AZ-861. Thus, it appears that the weaker antidepressant effect of AZ-861 may be due to its lower brain penetration.

The major disadvantages of antidepressant pharmacotherapy are SEs, which largely result from the blockade of histaminergic, cholinergic, and adrenergic receptors. The use of ADDs is often accompanied by weight gain, metabolic abnormalities, and cardiovascular effects, which are often the reason for premature discontinuation of antidepressant treatment. Therefore, in the further stage of our research, we assessed the safety profile of the studied compounds for these issues. We demonstrated that both AZ-853 and AZ-861 did not show muscarinic M_3_ receptor (M_3_R) antagonizing properties; thus, both AZ-853 and AZ-861 should not cause cholinolytic SEs. This is a very beneficial and desirable feature of these drugs, because SEs such as dry mouth, blurred vision, constipation, urinary retention, cognitive impairment, disorientation, and delirium are poorly tolerated by patients [51]. However, both compounds possess strong antihistaminergic effects. Additionally, AZ-853 blocked adrenergic α_1_ receptors (α_1_R) completely which resulted in decrease of methoxamine-induced increase in rat blood pressure, while AZ-861 showed weaker α_1_R antagonistic properties by reversing the effect of methoxamine by approximately 40%. Considering the histaminic H_1_ receptor (H_1_R) antagonizing and α_1_R-adrenolytic actions of the tested compounds, hypotension, sedation, and weight gain could be expected after their administration [52–54]. Indeed, AZ-853 significantly reduced diastolic blood pressure in normotensive anesthetized rats, whereas AZ-861 had no influence on rat blood pressure. The stronger impact of AZ-853 on blood pressure is not surprising, because its α_1_R antagonizing effect was over 2-fold more potent than that of AZ-861. Unfortunately, treatment with AZ-853 would therefore probably be associated with a higher risk of orthostatic hypotension, which may result in substantial morbidity and mortality from associated falls and syncope, particularly in elderly patients [55]. However, the hypotensive effect of AZ-853 was rather weak (13–15%) and observed at a dose many times higher than that active in FST (10 mg/kg vs. 0.625 mg/kg). It should also be noted that this compound did not affect diastolic blood pressure. Furthermore, there is evidence that the antagonism of α_1A_- and α_1D_-adrenoceptors results in antidepressant-like effects in mice [56]. Nevertheless, more experiments are needed to clarify this issue.

The effect of the tested compounds on daily spontaneous activity was monitored in animals housed in herds in their home cages; this approach allowed to avoid the effect of isolation-induced stress on mouse behavior. Compounds AZ-853 and AZ-861 administered repeatedly significantly decreased the spontaneous activity of animals, thus indicating their sedative potential. The sedative effect leading to decrease in caloric expenditure is postulated as one of the several mechanisms that may contribute to antidepressant-induced weight gain [3]. A slight but significant increase in weight was observed from the 10th day of AZ-853 administration as compared to that in the control group. In contrast, treatment with AZ-861 induced only transitory (from the 2nd to the 4th day of treatment) decrease in body mass and, in general, had no effect on mouse body weight. As histamine regulates feeding and obesity through hypothalamic H_1_R [52] and both compounds showed almost identical anti-histaminergic activity *in vitro*, we presume that the reason for this effect may be the weaker penetration of AZ-861 into the CNS.

In general, both sedation and weight gain are unwanted effects of treatment. However, in some clinical situations, they can provide additional therapeutic benefits. For example, the sedative properties of some antidepressant, antipsychotic, or anxiolytic drugs are desirable when the underlying disorder occurs with psychomotor agitation and/or insomnia [57]. Similarly, for senile cachexia or severe appetite disorder, the increase in body weight may provide additional benefits. A potential favorable effect of mirtazapine-induced increase in appetite and weight has been shown in underweight elderly patients with comorbid depression [58].

There is evidence that some ADDs may deteriorate glucose homeostasis and increase the risk of diabetes mellitus as well as induce lipid metabolism disturbances. Most of these metabolic changes are associated with ADD-induced weight gain [59]. In the biochemical experiments, AZ-853 and AZ-861 did not influence the plasma glucose level, but significantly increased the concentrations of triglycerides and cholesterol. Considering the lack of effect of AZ-861 on mouse body mass, it does not seem that the observed changes resulted simply from weight gain; therefore, more experiments are needed to explain this issue.

Organ weight can be the most sensitive indicator of the effect of drug toxicity, as significant differences in organ weight between treated and control animals may occur in the absence of any morphological changes. Aptly, none of the tested compounds affected the weight of liver, kidneys, and heart, expressed as the percentage of total body mass. Moreover, the activity of ALAT and γ-GTP was not significantly changed after repeated administration of AZ-861, thus indicating a low risk of hepatotoxicity. It should be emphasized that due to the hepatotoxic potential, regular monitoring of liver function is recommended while treating patient with ADDs, particularly when using agomelatine, duloxetine, bupropion or nefazodone [3]. Our present study showed a several-fold increase in the activity of γ-GTP after repeated administration of AZ-853. This result deserves attention and suggests further research, e.g., on alkaline phosphatase activity. γ-GTP present in serum is mainly of hepatic origin [60]. In liver diseases, the increase in γ-GTP level strongly correlates with the increase in alkaline phosphatase level. This information is clinically important because it allows γ-GTP increase in liver diseases to be differentiated from that due to other causes, e.g., cardiovascular diseases [61]. Researchers have shown a significant association of γ-GTP with known risk factors for cardiovascular diseases, such as triglycerides, cholesterol, and body mass index. The inclusion of these data in statistical analysis confirmed the significance of γ-GTP as an independent prognostic factor of death from cardiovascular causes [60]. All this information emphasizes the importance of conducting further safety tests for AZ-853, because the repeated administration of this compound caused an increase in plasma triglycerides and cholesterol as well as an increase in body weight.

## Conclusions

In conclusion, the present findings demonstrate that AZ-853 and AZ-861 administered once and repeatedly in mice showed antidepressant-like activity, which is partially dependent on 5-HT_1A_R activation. Between the two compounds, AZ-853 showed more potent antidepressant effect, presumably due to better penetration into brain structures. We also demonstrated that the studied compounds, which structurally differ by one substituent and its placement in the phenyl ring, varied from each other not just in their antidepressant potency and pharmacokinetic properties but also in their SE profile. Both AZ-853 and AZ-861 blocked H_1_R, resulting in weak sedation after repeated administration, and both induced lipid metabolism disturbances without affecting serum glucose level. None of the compounds showed anticholinergic properties. AZ-853 showed a stronger α_1_R-adrenolytic effect, which was manifested by a decrease in systolic blood pressure. Moreover, the compound induced mouse weight gain and plasma γ-GTP activity as compared to AZ-861. The interesting comparative pharmacological profiles of AZ-853 and AZ-861 encourage to conduct further experiments to fully understand their mechanisms and differences in action.

## Supporting information

S1 Fig The activity of 5-HT (A), AZ-853 (B) and AZ-861 (C) at 5-HT_1A_R *in vitro* functional assays. Graphs represent dose-response curves for functional activity of compounds in ERK1/2 phosphorylation, β-arrestin recruitment and inhibition of cAMP production assays.
(TIF)

S1 Table Influence of acute administration of AZ-853, AZ-861 and WAY-100635 given alone or combined with AZ-853 or AZ-861 on spontaneous locomotor activity in mice. Locomotor activity was measured from 3 to 6 min, that is the time equal to the observation period in FST. Data represent mean ± SEM, n = 5–8 mice per group; one-way ANOVA followed by Bonferroni’s post hoc test; ns–nonsignificant.
(DOCX)

S2 Table Influence of repeated administration of AZ-853 and AZ-861 on spontaneous locomotor activity in mice. Locomotor activity was measured 24 h after 12^th^ administration of compounds during 4 min, that is the time equal to the observation period in FST. Data represent mean ± SEM, n = 8–10 mice per group; one-way ANOVA followed by Bonferroni’s post hoc test; ns–nonsignificant.
(DOCX)

S1 Data Spontaneous locomotor activity data after acute administration. Raw data acquired with the spontaneous locomotor activity test. The columns represent the number of movements measured from 3 to 6 min, that is the time equal to the observation period in FST. On the right, the descriptive statistics.
(XLSX)

S2 Data Spontaneous locomotor activity data after repeated administration. Raw data acquired with the spontaneous locomotor activity test. The column represents the number of movements measured from 3 to 6 min, that is the time equal to the observation period in FST. On the right, the descriptive statistics.
(XLSX)

S3 Data Pharmacokinetic data. Raw data acquired with the pharmacokinetic studies. The columns represent the concentrations of the tested compounds in plasma, hippocampus, striatum, and frontal cortex at seven time points (5, 15, 30, 60, 120, 240, 480 min). The first sheet contains data for AZ-853, and the second for AZ-861. On the right, the descriptive statistics.
(XLSX)

S4 Data Blood pressure data. Raw data acquired with the blood pressure measurement. The columns represent the values of systolic and diastolic blood pressure (SBP and DBP, respectively) measured at eleven time points (0, 5, 10, 15, 20, 30, 40, 50, 60, 70, 80 min). The first sheet contains data for AZ-853, and the second for AZ-861. On the right, the descriptive statistics.
(XLSX)

S5 Data Body mass data. Raw data acquired with the body mass measurement. The columns represent the body weights measured for consecutive 15 days. On the right, the descriptive statistics.
(XLSX)

S6 Data Spontaneous activity monitoring data. Raw data acquired with the spontaneous activity monitoring. The columns represent counts registered every hour from the 1st to the 18th hour after treatment. The first sheet contains spontaneous activity data measured after the 1st and the second sheet after the 15th administration of vehicle or the tested compounds. *—outlier values excluded from statistical analysis. On the right, the descriptive statistics.
(XLSX)
