# Supplementary figures and images for: Antidepressant-like activity and safety profile evaluation of 1H-imidazo[2,1-f]purine-2,4(3H,8H)-dione derivatives as 5-HT1A receptor partial agonists
Source: PLoS One. 2020 Aug 7;15(8):e0237196. doi: 10.1371/journal.pone.0237196 (PMC7413516; doi:10.1371/journal.pone.0237196)

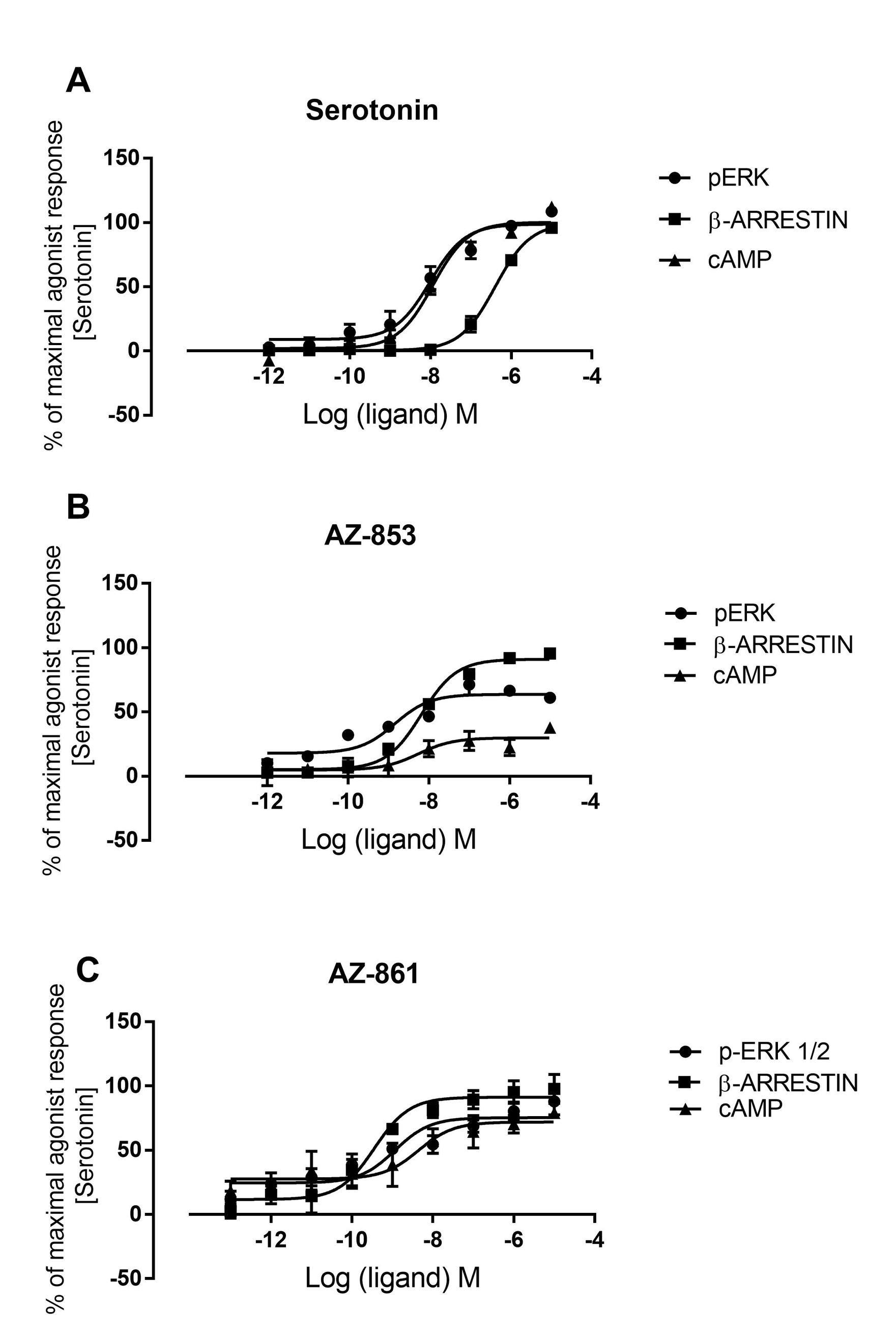

Supplement: S1 Fig — The activity of 5-HT (A), AZ-853 (B) and AZ-861 (C) at 5-HT1AR in vitro functional assays. Graphs represent dose-response curves for functional activity of compounds in ERK1/2 phosphorylation, β-arrestin recruitment and inhibition of cAMP production assays. (TIF) [file pone.0237196.s001.tif]
