# Supplementary material for: Antidepressant-like activity and safety profile evaluation of 1H-imidazo[2,1-f]purine-2,4(3H,8H)-dione derivatives as 5-HT1A receptor partial agonists
Source: PLoS One. 2020 Aug 7;15(8):e0237196. doi: 10.1371/journal.pone.0237196 (PMC7413516; doi:10.1371/journal.pone.0237196)
Supplement: S1 Table — Locomotor activity was measured from 3 to 6 min, that is the time equal to the observation period in FST. Data represent mean ± SEM, n = 5–8 mice per group; one-way ANOVA followed by Bonferroni’s post hoc test; ns–nonsignificant. (DOCX) [file pone.0237196.s002.docx]

| **Treatment** | **Dose (mg/kg)** | **Number of movements** | | |
| --- | --- | --- | --- | --- |
| **vehicle** | - | 168.1 | ± | 22.9 |
| **AZ-853** | 0.625 | 127.8 | ± | 25.0 |
|  | 1.25 | 149.8 | ± | 19.7 |
|  | 2.5 | 156.8 | ± | 32.0 |
|  |  | F(3, 21)=0,4623, ns | | |
| **AZ-861** | 1.25 | 167.3 | ± | 20.8 |
|  |  | F(1, 12)=0,0008, ns | | |
| **vehicle** | - | 112.9 | ± | 18.5 |
| **WAY-100635** | 0.3 | 122.9 | ± | 17.8 |
| **WAY-100635 + AZ-853** | 0.3 + 1.25 | 94.6 | ± | 12.6 |
| **WAY-100635 + AZ-861** | 0.3 + 1.25 | 97.4 | ± | 19.3 |
|  |  | F(3, 30)=0,5995, ns | | |
