# Supplementary material for: Antidepressant-like activity and safety profile evaluation of 1H-imidazo[2,1-f]purine-2,4(3H,8H)-dione derivatives as 5-HT1A receptor partial agonists
Source: PLoS One. 2020 Aug 7;15(8):e0237196. doi: 10.1371/journal.pone.0237196 (PMC7413516; doi:10.1371/journal.pone.0237196)
Supplement: S2 Table — Locomotor activity was measured 24 h after 12th administration of compounds during 4 min, that is the time equal to the observation period in FST. Data represent mean ± SEM, n = 8–10 mice per group; one-way ANOVA followed by Bonferroni’s post hoc test; ns–nonsignificant. (DOCX) [file pone.0237196.s003.docx]

| **Treatment** | **Dose (mg/kg)** | **Number of movements** | | |
| --- | --- | --- | --- | --- |
| **vehicle** | - | 508.0 | ± | 71.6 |
| **AZ-853** | 0.625 | 519.5 | ± | 59.8 |
| **AZ-861** | 1.25 | 513.9 | ± | 56.4 |
|  |  | F(2, 24)=0.0087, ns | | |
